# Metal-Organic Frameworks Applications in Synergistic Cancer Photo-Immunotherapy

**DOI:** 10.3390/polym15061490

**Published:** 2023-03-16

**Authors:** Pedro D. Fernandes, Fernão D. Magalhães, Rúben F. Pereira, Artur M. Pinto

**Affiliations:** 1 LEPABE, Faculdade de Engenharia, Universidade do Porto, Rua Roberto Frias, 4200-465 Porto, Portugal; 2 AliCE—Associate Laboratory in Chemical Engineering, Faculdade de Engenharia, Universidade do Porto, 4200-465 Porto, Portugal; 3 i3S—Instituto de Investigação e Inovação em Saúde, Universidade do Porto, Rua Alfredo Allen 208, 4200-135 Porto, Portugal; 4 INEB—Instituto de Engenharia Biomédica, Universidade do Porto, Rua Alfredo Allen 208, 4200-135 Porto, Portugal; 5 ICBAS—Instituto de Ciências Biomédicas Abel Salazar, Universidade do Porto, 4050-313 Porto, Portugal

**Keywords:** nanomaterials, cancer therapy, photodynamic therapy, photothermal therapy, immunotherapy

## Abstract

Conventional cancer therapies, such as radiotherapy and chemotherapy, can have long-term side effects. Phototherapy has significant potential as a non-invasive alternative treatment with excellent selectivity. Nevertheless, its applicability is restricted by the availability of effective photosensitizers and photothermal agents, and its low efficacy when it comes to avoiding metastasis and tumor recurrence. Immunotherapy can promote systemic antitumoral immune responses, acting against metastasis and recurrence; however, it lacks the selectivity displayed by phototherapy, sometimes leading to adverse immune events. The use of metal-organic frameworks (MOFs) in the biomedical field has grown significantly in recent years. Due to their distinct properties, including their porous structure, large surface area, and inherent photo-responsive properties, MOFs can be particularly useful in the fields of cancer phototherapy and immunotherapy. MOF nanoplatforms have successfully demonstrated their ability to address several drawbacks associated with cancer phototherapy and immunotherapy, enabling an effective and low-side-effect combinatorial synergistical treatment for cancer. In the coming years, new advancements in MOFs, particularly regarding the development of highly stable multi-function MOF nanocomposites, may revolutionize the field of oncology.

## 1. Introduction

Cancer remains a growing concern, not only in terms of global public health but also as a social and economic issue. According to GLOBOCAN, 19.3 million new cases of cancer were estimated in 2020, accounting for 10 million deaths globally. The number of diagnoses is expected to rise to 28.4 million by 2040 [1]. Therefore, the search for more effective solutions to this problem is increasingly important. Currently, the most commonly employed cancer treatments include surgery, radiotherapy, and chemotherapy [2]. However, such treatments have a variety of downsides and side effects. Because of their poor distribution at the tumor site and the high concentrations required, administered drugs have a substantial limitation in cancer treatment, leading to cumulative multidrug resistance [2,3]. Furthermore, high dosages of chemotherapeutic agents and high-intensity radiation can have nefarious effects on adjacent healthy tissues [3]. As a result, these limitations encourage research for more targeted and low-side-effect methods, including phototherapy and immunotherapy [4,5].

Phototherapy is a minimally invasive and highly selective treatment that involves the incidence of a light beam onto a specific region while minimizing the adverse effects on healthy tissues [6]. Cancer phototherapy consists of killing tumor cells by the action of phototherapeutic agents under light irradiation. There are two widely studied strategies for phototherapy: photodynamic therapy (PDT) and photothermal therapy (PTT) [7].

Photodynamic therapy (PDT) is a therapeutic approach based on the conversion of light into chemical energy [6]. When a specific wavelength of light irradiates a photosensitizer (PS), it absorbs the energy and becomes excited or activated, triggering a series of photochemical reactions that produce highly reactive oxygen species (ROS), such as superoxide anion radical (˙O_2_^−^), hydroxyl radical (˙OH), hydrogen peroxide (H_2_O_2_) in type I or electron transfer reaction, and singlet oxygen (^1^O_2_) in type II or energy transfer reaction in type II or energy transfer reaction [7,8,9]. ROS oxidation of biomolecules such as lipids, proteins, and DNA has a cytotoxic effect on tumor cells by impacting cell signaling cascades and/or gene expression regulation [9]. In contrast to conventional therapies, in PDT, due to the selectivity of the PS for tumor cells, it only accumulates within the malignant tissue, while the irradiation area is limited to the tumor site [10]. However, there are also several limitations to this technique in terms of PS photochemical and physiological properties, as well as light settings and cancer tissue characteristics [11]. These are related to their hydrophobicity, low photodynamic yield, insufficient pharmacokinetics, and low selectivity for malignant tissues. However, these obstacles can be overcome using nanomaterial-designed delivery systems that improve PDT efficiency [11,12].

PTT, as with PDT, is a minimally invasive and selective therapeutic approach that uses photothermal agents (PTAs) to absorb light and convert it into thermal energy or heat, resulting in the thermal ablation of cancer cells [8,13]. Light energy absorption causes the PTAs to be excited from their ground state to a singlet excited state, which is then converted to thermal energy via a non-radiative vibrational relaxation induced by intramolecular movements and collisions with surrounding molecules, increasing kinetic energy and, consequently, temperature [8,14]. The increase in cell temperature causes enzyme release and cell lysis, leading to cell necrosis, protein denaturation, and cancer cell death [13]. Some photothermal agents have significant drawbacks, including high cost, poor photothermal stability, low photothermal conversion efficiency, and the possibility of toxicity and adverse effects [15].

Due to the newfound capability to trigger immunogenic cell death (ICD), phototherapies have become even more appealing. When subjected to excessive physicochemical or mechanical stress, tumor cells undergo an apoptotic state, prompting multiple events with the release of tumor-associated antigens (TAA) and the presentation of several damage-associated molecular patterns (DAMPs), such as increased exposure of the chaperone calreticulin (CRT), associated to a protein unfolding response, the release of high-mobility group box 1 (HMGB1), and adenosine triphosphate (ATP) secretion, eliciting an immunomodulatory activity and long-lasting immune response [16,17,18,19,20,21]. Simultaneous release of several DAMPs is critical for dendritic cell (DC) maturation as well as for innate and adaptative immune responses [22]: (i) CRT exposure functions as a “eat me” signal for tumor cell phagocytosis, while also promoting DC cell maturation by inciting the production of the cytokines interleukin 6 (IL6) and tumor necrosis factor (TNF), for CD4^+^ T helper cell (Th17) polarization [22,23]; (ii) ATP secretion functions as a short-range “find me” signal for DCs, that aids DC activation while further promoting the secretion of cytokine IL-1β, crucial for the activation of T cell-dependent antitumor immunity [22,24]; (iii) the binding of released HMGB1 to the toll-like receptor (TLR)-4 on DCs increases the production of pro-inflammatory cytokines and enhances antigen presentation, while simultaneously suppressing immunosuppressive regulatory T (Treg) cells [22]. Immune activation is further promoted by the engulfment of TAAs by DCs, which are then presented on the cell surface as major histocompatibility complex (MHC) molecules I and II to activate “naive” T lymphocytes [25]. The induction of localized inflammation is of utmost importance in the treatment of patients with nonimmunogenic or “cold” tumors. The infiltration of immune cells such as macrophages, neutrophils, natural killer (NK) cells, DCs, and lymphocytes can reverse the immunosuppressed tumor microenvironment (TME) and turn “cold” (non T cell inflamed) into “hot” (T cell inflamed) tumors [16,25,26]. Despite being regarded as a potential cancer therapy modality, the strength of the induced ICD is affected by several factors, including the low efficiency of PS, hypoxic TMEs, and low ROS accumulation during PDT, or the limited efficiency of photothermal agents with good biocompatibility in PTT. As a result, single-modal immunotherapy based on phototherapy-induced ICD is insufficient to elicit a robust immune response [27,28,29].

Immunotherapy has been widely used to treat cancer in the past few decades [30]. Unlike other conventional therapies that attempt to suppress or prevent tumor growth or proliferation, immunotherapy uses biotherapeutics to enhance the natural defenses of the immune system, reducing the tumor-induced immunosuppression and triggering an antitumor immune response that not only suppresses primary tumor growth but also prevents metastasis and tumor recurrence [29,30,31]. Immune checkpoint blockade (ICB), adoptive T cell therapy (ACT), and chimeric antigen receptor (CAR) T cell treatment have lately drawn significant attention for their ability to directly target the tumor microenvironment, activating tumor-specific T cells and cytotoxic T cells for an effective immune response against cancer [31,32]. Despite the substantial reduction in side effects when compared to other types of therapies, the unpredictability of immune-related adverse events (irAEs) in different organ systems, the cytotoxicity associated with the treatment, and resistance to the therapy are all concerning factors in the administration and efficacy of immunotherapy [31,32,33]. Furthermore, due to the lack of tumor-specific antigens (TSAs) and the immunosuppressive environment of the TME, immunotherapy effects on solid or “cold” tumors are limited [32,34]. To address the shortcomings of the different therapies, multimodal strategies that combine phototherapy-induced ICD and immunotherapy can be employed to improve cancer treatment [29].

**Figure 1:**
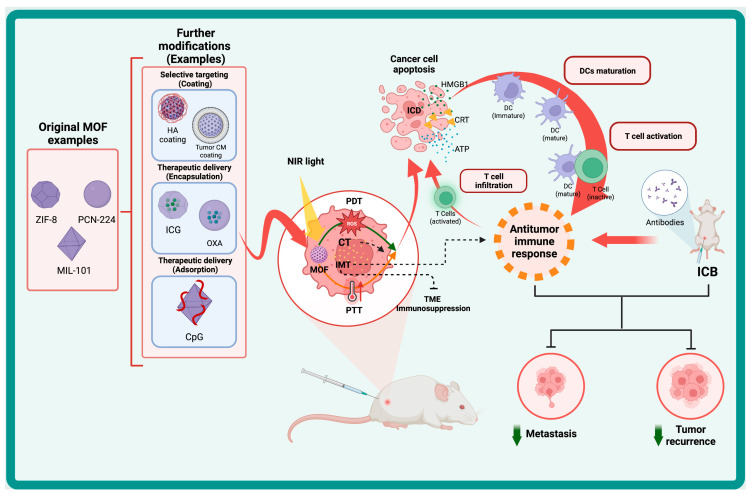
Metal-organic frameworks (MOFs) types, modifications, and synergistic cancer photo-immunotherapy mechanisms. Commonly used MOFs for biomedical applications, such as ZIF-8, PCN-224, and MIL-101, can be modified in a rational design for selective targeting and function as nanocarriers for therapeutic drug delivery (e.g., immune adjuvants (CpG), chemotherapeutic agents (OXA) and photothermal agents (ICG)) into the TME. The incidence of NIR light in tumor cells containing the MOF can trigger either PDT or PTT depending on the intrinsic properties of the MOF or the photosensitizer loaded. The intracellular environment of the tumor, as well as the NIR light, can trigger the release of chemotherapeutic and immunotherapeutic agents that can synergize through ROS production or immunotherapeutic effects. PTT and PDT-induced ICD triggers the exposure of CRT, HMGB1 release, and ATP secretion, subsequently inducing DCs maturation. DCs present tumor antigens to naïve T cells, activating them and triggering an antitumor immune response, consequently increasing the infiltration of T cells at the tumor site. Synergetic therapy with ICB promotes an enhanced systemic antitumor immune response, conferring protection against metastasis and tumor recurrence. Abbreviations: HA, hyaluronic acid; CM, cell membrane; ICG, indocyanine green; OXA, oxaliplatin; CpG, cytosine-phosphate-guanine; NIR, near-infrared region; MOF, Metal-organic framework; PDT, photodynamic therapy; PTT, photothermal therapy; IMT, immunotherapy; CT, chemotherapy; TME, tumor microenvironment; ICD, immunogenic cell death; DC, dendritic cell; ICB, immune checkpoint blockade. Created with BioRender.com.

Cancer nanomedicine has emerged with a wide range of organic, inorganic, or organometallic nanoparticles that function as efficient platforms for cancer imaging, diagnostic, therapeutic, and theranostic strategies [35]. Several platforms based on nanomaterials have been created, with the most common formulations being liposomes, micelles, nanocrystals, polymers, dendrimers, two-dimensional (2D) materials, nanotubes, and core/shell nanoparticles [36,37]. To address the limitations of different cancer therapies, nanotherapeutic techniques, based on the properties of nanomaterials, were devised to control drug release, kinetics, and pharmacodynamics, resulting in a safer and more efficient cancer treatment [36]. Several nanoplatforms have been developed for synergistic photo-immunotherapy, which can modulate immune response by themselves or serve as carriers for different therapeutics and immunotherapeutic agents for delivery to a specific target at the same time [38,39]. Upconversion nanoparticles (UCNP), gold nanoparticles (Au NP), copper sulfide nanoparticles (CuS NP), Prussian blue nanoparticles (PBNP), carbon nanomaterials, and metal-organic frameworks (MOFs) are examples of basic nanomaterials used in synergistic photo-immunotherapy [39]. Among these nanomaterials, the properties of MOFs provide significant advantages for a wide range of biological applications, including photo-immunotherapy [40,41]. Indeed, the large surface area, porous structure, and flexibility of the coordination between organic ligands and nodes enable the development of suitable MOF-based delivery systems that may be modified for a variety of therapeutic purposes while maintaining good biocompatibility [41,42]. MOFs may also be synthesized with functional nodes and linkers that have inherent antitumor and photosensitizer properties [41]. MOFs have sparked the interest of researchers in biomedical fields in recent years. Until now, only a few excellent reviews regarding the use of MOFs in immunotherapy and phototherapy for cancer have been published [7,43,44]. However, the use of MOFs in synergetic photo-immunotherapeutic approaches is poorly discussed in literature. This review intends to provide a thorough overview of MOF applications and their potential use as nanoplatforms for synergetic photo-immunotherapeutic approaches in cancer therapy (Figure 1). The first section aims to present the structure, properties, and applications of MOFs in different fields. The roles of MOFs as intrinsic photosensitizers and photothermal agents or nanocarriers of exogenous photosensitizers (PSs), as well as photothermal agents (PTAs) used in photodynamic and photothermal therapies against cancer, will then be discussed. The second section will highlight the latest strategies for using MOFs as nanoplatforms for synergistic photoimmunotherapy in cancer. The last section will aim to address the primary challenges and opportunities in the field and draw conclusions from presented literature.

## 2. Metal-Organic Frameworks (MOFs)

### 2.1. Structure and Properties

**Figure 2:**
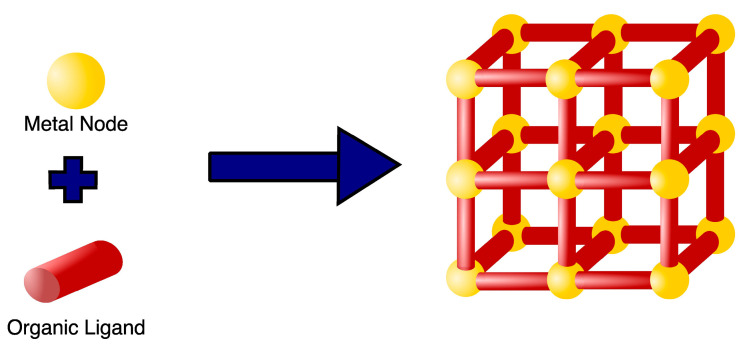
Example of a basic MOF structure. MOFs are highly organized porous nanomaterials composed of metal nodes coordinated with several organic ligands serving as a bridge between nodes.

MOFs are a class of highly organized porous nanomaterials with a crystalline inorganic-organic hybrid structure, assembled from multiple coordination of organic linkers and inorganic metal ions as cluster nodes (Figure 2) [45,46]. The inorganic components of MOFs, known as secondary building units (SBUs), may contain a variety of alkaline earth metals, alkali metals, transition metals, actinides, lanthanides, and several main groups of metal ions that are primarily in carboxylate form to coordinate with a variety of organic ligands (bipyridyl, imidazolate, and carboxylate-based) and biological macromolecules (amino acids, peptides, nucleobase, and saccharide) [47]. Organic linkers act as bridging ligands between metal nodes, with di-, tri-, and tetra-carboxylate ligands (e.g., Terephthalic acid, 2-aminoterephthalic acid, benzene tricarboxylic acid (BTC) and trimesic acid) being commonly used due to their sterically rigid and highly polarized aromatic structures, allowing for complex morphologies as well as more rigid frameworks [48,49]. In contrast to other porous nanomaterials, such as zeolites and carbons, the MOFs structure can be tailored to the desired application, since SBUs geometry and the size and shape of organic ligands are determinant structural factors that can be selected to achieve the desired pore size, structure, and function [50]. Recently, researchers have been building more complex MOFs, or highly organized meso- and macroscopic superstructures, using nanocrystals as building blocks, exploiting the different metal-ligand geometries (tetrahedral, octahedral, and cubic) [51,52,53]. Such complex superstructures are classified into four dimensions: (i) zero-dimensional (0D) in the form of hollow capsules or microspheres; (ii) one-dimensional (1D) as nanorods and nanofibers; (iii) two-dimensional (2D) nanostructures of platelets, sheets, plates, films, and membranes; and (iv) three dimensional (3D) nanostructures as an extension of 0D, 1D, and 2D superstructures across multiple length scales [52,53,54].

In recent decades, MOFs have emerged as intriguing nanotechnology materials due to their potential in a wide array of applications, including gas storage, chemical separation, catalysis, magnetism, sensing and detection, drug delivery, and other biomedical applications such as cancer therapy, osmotic and diffusion-controlled membranes, tissue engineering, gasotransmitter therapies, biosensing, bioimaging, biocatalysts, and antibacterial [55,56,57,58,59,60]. Many MOFs, for example, have previously been developed for biomedical applications in the domains of bone treatment and bone repair, such as Cu-TCPP-TCP for bone tumors, ZIF-8/VAN for osteoarthritis, and Zr-MOFs for bone regeneration [61]. Titanium MOFs (Ti-MOF) are yet another type of MOF that has been developed for biomedical applications, including antibacterial, anti-inflammatory, bone damage, and cancer therapy [62]. MOFs have unique properties that cannot be found in organic or inorganic systems due to their hybrid nature [63]. MOFs comprising different metal ions and organic linker structures feature different morphologies, pore diameters, and unique electrical, magnetic, and optical properties that can be used in specific applications [50,55]. One of the most appealing properties of MOFs as a basis for their functions is their constant highly organized porosity. Until recently, the majority of MOFs developed have been microporous (<2 nm) with a large surface area imparting good adsorption of various gases such as hydrogen and carbon [50,64]. However, this pore size is unsuitable for other applications, such as catalysis and drug delivery, that require mesoporous (2–50 nm) and microporous (>50 nm) MOFs with a larger surface area [50,65]. The linear extension of organic linkers tends to be a solution to provide large storage space and a higher number of adsorption sites within the crystal framework. The increased space between the pores, may stimulate the formation of interpenetrating structures (the intertwined growth of two or more frameworks) [64]. As a result, the synthesis of mesoporous and macroporous MOFs remains a problem for the various applications of MOFs [50].

In addition to the high porosity and large surface area, other properties, such as easy functionalization, inherent biocompatibility, water solubility, biodegradability, and thermal stability, aroused the interest in MOFs, particularly as drug delivery systems (DDS) [66,67]. Since MOFs are tunable, they can accommodate a wide range of molecules with varying physicochemical properties that can be incorporated into the MOF via surface attachment, covalent bonding, pore encapsulation, and in situ encapsulation through multiple interactions with the linkers (e.g., hydrogen bonds, electrostatic interactions, van der Waals forces, stacking, covalent bonds, and coordination bonds) [55,67]. Additionally, due to constant porosity, these flexible network structures are stimuli-responsive under stress, changing their properties and/or structures in different environments [63,68]. MOFs’ inherent ability to undergo a molecular change in response to specific stimuli allows for controlled induction in a desired environment for several applications [68]. MOF transformation can be triggered by the presence of specific endogenous environments (pH, ATP, redox, glutathione (GSH)) or by the reaction to external stimuli (different wavelengths of light, temperature, pressure, magnetic field, ions, and humidity) [68,69]. Stimuli-responsive MOFs can act as delivery systems for several bioactive molecules chemotherapeutic agents, fluorescent agents, and organic dyes for application in chemotherapy, biomedical imaging, PDT, and PTT, enhancing their efficiency and potentially diminishing side effects [68,70,71]. As an example, Qin et al. developed a novel hydrostable 2D Zn-based MOF as drug delivery system for 5-fluorouracil (5-FU), a typical anticancer drug. 5-FU encapsulation in the MOF could potentially inhibit poor biodistribution, as a release assay, reports a slow release of 5-FU with no bursting effects. Moreover, cytotoxicity, evaluated by a 3-(4,5-dimethyl-2-thiazolyl)-2,5-diphenyl-2-H tetrazolium bromide (MTT) assay, displayed >90% cell survival [72]. 

Despite the promise promoted by their distinct properties, early MOFs, primarily developed using divalent metals such as Cu^2+^ and Zn^2+,^ proved unsuitable for certain applications due to stability issues under harsh conditions (e.g., moisture or aqueous solutions), limiting their application and commercialization [73,74]. Several factors, including metal ions and organic ligand composition, metal-ligand coordination geometry, pore surface hydrophobicity, and the operating environment (e.g., the presence of water, temperature, pH, and pressure), have been reported to affect MOF stability, resulting in poor water stability, acid/base stability, thermal stability, and mechanical stability [50,74,75]. The rationalization of MOFs stability under certain conditions is critical for employment in the desired application. As a result, the consideration of metal-ligand bond strength and various kinetic parameters is critical for the development of more stable MOFs [50,76]. In recent years, several solutions have been developed to tackle different stability problems, including increasing the strength of coordination bonds and the surface hydrophobicity of MOFs for better water stability, combining high oxidation-state metal ions or hard acids with carboxylate linkers (hard bases) to generate strong bonds and increase acid/base stability, and using high valence metal ions (e.g., Ln^3+^, Al^3+^, Zr^4+^, and Ti^4+^) to achieve higher thermal stabilities [50]. Although studies on improving mechanical stability are scarce, the functional groups of organic ligands appear to have an impact MOF mechanical stability [50,77]. Recent advances in MOF-hydrogel composites may provide a solution for improving MOF stability, not only for biomedical applications but also in other sectors [78]. Nonetheless, a better understanding of the factors influencing structural stability has resulted in the growing development of more stable MOFs and the expansion of many applications [50].

### 2.2. MOFs in Phototherapy

Phototherapy uses near-infrared region (NIR) light to kill cancer cells by generating ROS in PDT and inducing thermal ablation in PTT [79]. The selection of the best PSs or PTAs in both therapies has a significant impact on the therapy’s efficacy [80]. Ideal photosensitizers are non-toxic or have minimal toxicity, display high absorbance in the NIR wavelength, and exhibit high photostability [81]. Despite the development of several inorganic materials and nanoparticles (e.g., noble metals, semiconductors, carbon nanomaterials, magnetic nanoparticles, and manganese dioxide) and organic compounds (e.g., indocyanine green and porphyrin) as photosensitizers, the in vivo non-biodegradability, high toxicity, possible long-term toxicity of inorganic nanoparticles, and easy photobleaching of organic compounds limits their phototherapeutic applications [7,81]. Furthermore, other drawbacks, such as hydrophobicity-induced aggregation, limited diffusion of ROS, oxygen dependence, undesirable penetration depth in PSs, and limited penetration depth and lack of selectivity in PTAs, highlight the need for improvement and the development of more elaborate phototherapeutic strategies [7].

Over the last decade, there has been an increasing interest in the intrinsic photodynamic and photothermal capabilities of certain MOFs [7]. Another appealing feature is that the porous structure of MOFs enables the loading of phototherapeutic agents for photo-responsive release, preventing self-aggregation and self-quenching of PSs and improving photothermal responses and thermal stability of PTAs [7,81]. Furthermore, the nanomaterials’ superior biocompatibility, ease of modification, passive targeting of enhanced permeability and retention (EPR), and TME-responsive degradation make them attractive candidates for enhanced phototherapy treatments [81]. On the other hand, MOFs demonstrate difficulties in adapting to the TME due to poor water stability. However, by using core-shell structures, where MOFs may act as the core or shell that binds to other materials, it is possible to solve stability issues while keeping the original functional capabilities. Examples of those other materials includes metal oxides, organic polymers, and carbon nanoparticles [7].

PDT is a novel and non-invasive therapy that specifically destroys tumor cells; it is dependent on the efficiency of the photosensitizer, light, and oxygen available in the TME [81]. Synthesis of intrinsic photodynamic MOFs often involves the use of porphyrins and their derivatives (dihydroporphyphenol, chlorophyllin) [7,82]. Porphyrins are organic heterocyclic macrocycles composed of four pyrrole groups connected by methylene bridges [82,83]. Their application in phototherapy is attractive due to their prevalence in natural systems; this makes them ideal for use in biological singlet oxygen production with the absence of significant cytotoxicity without light [84]. Furthermore, porphyrin has 22 π-electrons of which 18 are conjugated, facilitating π–π* transitions to yield a Soret, or B band at ~400 nm (electronic transition from the ground state to a second excited singlet state (S_0_ → S_2_)) and four lower energy and low-intensity Q-bands between ~450 and 650 nm (S_0_ → S_1_) [83]. Porphyrin derivatives with fewer π-electrons show increased red-shift absorptivity Q-bands at wavelengths ranging from 650 to 800 nm [7,83]. The overlap of absorption in the red with the highest tissue penetration region raises interest in PDT applications [84]. High hydrophobicity and aggregation, on the other hand, restrict bioavailability and accumulation at target sites, limiting their therapeutic applicability [83]. The use of porphyrin-based MOFs improves PS efficiency by preventing aggregation and enhancing ROS diffusion due to the porous structures of the MOFs. Therefore, several porphyrin-based MOFs have been developed for application in PDT [7].

Lu et al. created the first porphyrin-based MOF in a rational design of a hafnium (Hf)-porphyrin nanoscale MOF. DBP-UiO MOF (DBP referring to dibenzoporphyrin and UiO referring to University of Oslo) was created via a solvothermal reaction involving HfCl_4_ and the porphyrin derivative, 5,15-di(p-benzoato) porphyrin (H_2_DBP), originating a UiO-type MOF crystal structure composed of hexanuclear clusters of SBU twelve-fold bonded to bridging ligands. Through the isolation of the photosensitizer, which prevents aggregation and self-quenching, and the enhancement of intersystem crossing by the heavy Hf center, DBP-UiO efficiently boosted ROS formation and, as a result, PDT efficacy. In vivo studies revealed that half of the treated mice exhibited tumor volume reduction while the other half experienced tumor eradication, emphasizing the significant promise of nanoscale MOFs (nMOFs) as strong PDT agents [85].

In addition to Hf, other metal centers, such as manganese (Mn) and, most commonly, zirconium (Zr), can be used for the construction of MOFs with intrinsic photodynamic properties [86]. Among several MOFs developed, the porous coordination network (PCN) family, consisting of Zr_6_-based porphyrinic MOFs with high surface areas, is particularly important for structurally guided strategies in photodynamic therapies [86,87,88]. Park et al. developed size-controllable PCN-224 through a solvothermal reaction of 6 Zr_6_ clusters (primarily octahedral) coupled to a tetrakis(4-carboxyphenyl)-porphyrin (TCPP) ligand into a spheric morphology. A size-controlled PCN-224 might increase cellular uptake and, as a result, PDT efficiency, highlighting the relevance of size parameters of the nanoplatforms in cellular response [89]. Furthermore, post-synthetic modifications of MOFs can be an efficient method of increasing PDT efficacy. Porphyrin MOF surface modification approaches have included cell-penetrating peptide, folic acid (FA), hyaluronic acid (HA), erythrocyte membrane, cancer cell membrane, exosome, metal nanoparticles, and nano enzymes, to name a few [86,90,91,92,93,94,95,96,97]. According to Park et al., further functionalization with FA in PCN-224 enabled active targeting of tumor cells and further improved PDT performance [89].

Non-intrinsic photodynamic MOFs can also be employed in PDT by incorporating PSs into the MOF structure through encapsulation, surface attachment, or the construction of a core-shell structure. MOF alternatives are more diversified without the constraint of porphyrins and their derivatives, including ZIF-8 (zeolitic imidazolate framework), MIL-101 (Materials Institute Lavoisier), and UiO-66, often used in biomedical applications [7,98]. ZIF-8 consists of a robust 3D network composed of tetrahedral zinc (Zn) ions connected by 2-methyl imidazolate ligands, normally with a sodalite topology [99]. Zheng et al. reported a pH-responsive ZIF-8-based nanoplatform by incorporating gold nanoclusters (AuNCs) as photosensitizer and doxorubicin (DOX) as a chemotherapeutic agent for a PDT/chemotherapy synergistic therapy. Under the acidic conditions of the TME, the ZIF-8 structure is destroyed promoting the delivery of both AuNCs and DOX for an enhanced PDT/Chemotherapy therapeutic effect [100]. MIL-101, on the other hand, is composed of a metal-(III) trimer consisting of three octahedra that laterally bind to two carboxylic groups of two terephthalic acids [1,4-benzene dicarboxylate (H_2_BDC)] molecules, culminating in a super tetrahedron topology. MIL-101 has an extraordinarily large surface area and pore volume, as well as good air, water, and acid stability [101,102]. In a strategy for PDT target switching, Liu et al. modified MIL-101(Fe) with amino groups (NH_2_) for surface attachment of the photosensitizer chlorine e6 (Ce6)-labeled cathepsin B (CaB) substrate peptide. The MOF composite was then loaded with a camptothecin anticancer agent for a combined PDT and chemotherapy treatment. The transfer of the excited electron to the MOF hindered the fluorescence of Ce6. When Ce6 came into contact with CaB in TME, it was cleaved off of the MOF surface, regaining its fluorescence and the ability to activate PDT for effective combined cancer therapy [103]. UiO-66 is a conventional MOF composed of Zr^4+^ ions as metal nodes coupled by terephthalic acid molecules that exhibits desirable drug carrier properties, including a large surface area, physicochemical stability, and low toxicity [98,104]. Ding et al. designed a novel multifunctional MOF for a PDT/chemotherapy synergistic antitumor treatment through the functionalization of UiO-66-NH_2_ with the encapsulation of 5-aminolevulanic acid (ALA-5), a protoporphyrin precursor, as a photosensitizer and the formation of a core-shell structure promoted by the affinity of pemetrexed (MTA) (a chemotherapeutic agent that possesses high antitumor activity and targeting ability as a folate antagonist) to the unsaturated Zr active site of UiO-66-NH_2,_, reaching high loading rates. MTA's greater affinity for folate receptors improved tumor cell targeting and uptake. Moreover, an effective PDT/chemotherapy combination therapy remarkably suppressed tumor growth [104]. 

PTT, like PDT, is a new and non-invasive therapy that uses the conversion of light energy into heat energy to increase the temperature and achieve therapeutic effects at the lesion site [105]. PTT can be directly mediated by MOFs with inherent photothermal properties without the introduction of an exogenous PTA by using several PTAs as ligands (e.g., IR825 and ferrocene (Fc)) [7]. In this regard, Yang et al. designed a self-assembling MOF with Mn^2+^ as the metal node and PTA, IR825, as the ligand to achieve excellent NIR absorbance and photothermal stability. To improve biocompatibility, the nanoparticles were further modified with polydopamine (PDA) and polyethylene glycol (PEG), generating Mn-IR825@PDAPEG nanoscale metalorganic particles (NMOPs). Under 808 nm light irradiation, the NMOPs demonstrated strong photothermal performance and effective tumor ablation [106]. In another example, Deng et al. built a Zr-Fc MOF nanosheet for a PTT/CDT synergetic method. Zr clusters were bridged by 1,1-ferrocenedicarboxylic acid [Fc(COOH)_2_] ligands in the Zr-Fc MOF, resulting in a nanosheet with excellent light absorbance and good photo-thermal conversion efficiency (PCE). Additionally, Zr-Fc MOF has endowed a Fenton catalytic activity from the Fc ligand that converts H_2_O_2_ into the hydroxyl radical (^•^OH), for an additional chemotherapeutic effect. The combined action of PDT and chemotherapy led to the death of >80% of 4T1 tumor cells in vitro under 808 nm irradiation for 3 min and nearly 100% after 5 min. Furthermore, in vivo tumor growth was effectively suppressed, suggesting a viable MOF-based nanoplatform with the potential for PTT cancer therapy without the use of exogenous PTAs [107].

Prussian blue (PB) is a MOF archetype that has been authorized as a clinical antidote for internal radioactive contamination by the United States Food and Drug Administration (FDA) [108,109]. PB is a coordination polymer with a cubic porous network structure composed of ferric ions (Fe^III^) and ferrous ions (Fe^II^) coupled to a nitrogen atom and carbon atom of a cyanide molecule that bridges both iron ions, assuming an ideal formula of Fe^III^_4_[Fe^II^(CN)_6_]_3_nH_2_O [109]. PB has been widely employed in PTT as a MOF with inherent photothermal capabilities due to its strong light absorption and photo-thermal conversion efficiency in the NIR. NIR light is converted into heat by electron migration between Fe III and Fe II via the cyanide ligand, promoting therapeutic hyperthermia [109,110,111]. Furthermore, PB has minimal biotoxicity and good biodegradability for biomedical applications [110]. Peng et al. established a simple, low-cost, and environmentally friendly approach to synthesize carbon dot (CD)-decorated Prussian blue nanoparticles (CDs/PBNP) nanocomposites, combining CD photoluminescent capabilities with PBNPs photothermal conversion ability. CD/PNBP presented high photothermal conversion efficiency (30%) and photothermal stability, evoking an efficient photothermal cytotoxic effect on C6-tumor-bearing mice subjected to light irradiation at 808 nm for 10 min [112].

In a similar fashion to PDT, exogenous PTAs can also be incorporated into several MOFs (e.g., ZIF-8 and MIL-100) by encapsulation in the porous structure or as either the core or shell of a core-shell MOF structure [70]. As an example, Tian et al. reported the encapsulation of graphene quantum dots (GQD) and of the chemotherapeutic agent DOX into ZIF-8 to generate DOX-ZIF-8/CQD nanoparticles for a controlled drug delivery system in a synergistic therapeutic approach. GQD provided the nanoparticles significant NIR absorbance, PCE, and outstanding thermal conductivity for a good photothermal effect while also endowing the capability to adjust the therapeutic temperature through NIR intensity, time of irradiation, and DOX-ZIF-8/GQD nano-particle concentration. Furthermore, CQD dissociation increased ZIF-8 pH-sensitive DOX release. As a result, the synergistic impact of chemo- and photothermal treatment, as well as the improved delivery mechanism, demonstrated the development of a multifunctional nanoplatform with the potential for effective cancer cell ablation [113]. In another study, Fan et al. developed PPy@MIL-100 core-shell nanoparticles in a synergistic PTT/chemotherapeutic strategy, making use of polypyrrole’s (PPy) high photothermal conversion efficiency and excellent biocompatibility. The nanoparticles were synthesized with PPy as the core coated by an iron (III) carboxylate MOF (MIL-100) outer shell, which was then loaded with DOX anticancer drug. Under 808 nm light irradiation, the nanoparticles displayed an improved pH and NIR-responsive drug release, as well as a photothermal effect for enhanced tumor cell cellular death [114].

## 3. Synergistic Photo-Immunotherapy

Phototherapies can act as the first line of defense against cancer, killing primary tumors in an effective and non-invasive manner. Phototherapy’s capacity to induce ICD has attracted a great deal of attention due to its potential application in cancer therapy by converting “cold” tumors into “hot” ones [115,116,117]. However, due to the low efficiency of ICD, phototherapy’s capacity to trigger immune response is typically limited [116,117]. Immunotherapy, on the other hand, can instruct the immune system to recognize and destroy tumor cells, preventing tumor recurrence, but the low targeting specificity of these therapies might cause adverse immunological effects in patient’s organs [115,118]. As a result, combining phototherapy with immunotherapy into photo-immunotherapy (PIT) and synergetic photo-immunotherapy is a win-win method for optimized cancer therapy with minimum side effects [115].

In recent years, MOFs have attracted attention for their potential to serve as nanoplatforms for PIT and synergetic photo-immunotherapy, owing to their unique versatility and properties that allow them to function as photothermal or photodynamic agents, as well as nanocarriers for immunotherapeutic and phototherapeutic therapies [7,119]. Table 1 summarizes the outline of the application and efficacy of MOFs in different PIT and synergetic photo-immunotherapy strategies for cancer therapy.

### 3.1. Synergistic Strategies of PDT and Immunotherapy

PDT is a therapeutic approach in which a photosensitizer, triggered by light, releases energy to produce ROS, resulting in the non-invasive ablation of cancer cells. The pressure exerted on the endoplasmatic reticulum by ROS accumulation exposes CRT and triggers ICD, hence promoting an antitumor immune response [145,146,147]. However, because PDT efficiency is affected by the light source, the PS, and the level of oxygen available in the TME, a low efficient buildup of ROS results in a weak ICD. Problems such as the PS half-life and self-quenching, the low depth of light penetration through biological tissues, and the hypoxic conditions of the TME severely hinder ROS production and thus ICD efficiency, rendering it insufficient to elicit an immune response and attain the desired therapeutic effects [29,146]. As a result, PDT combined with other therapies, such as immunotherapy (e.g., immune checkpoint blockade and immune adjuvants), complements the shortcomings and maximizes the strengths of each treatment, potentially producing synergistic results [148,149]. As described in previous sections of this review, MOFs can function as a nanoplatform for the combination of PDT with other treatments, allowing the best of both therapies to be exploited. The introduction of organic ligands with photodynamic characteristics, such as porphyrin ligands, into MOF structures, as well as the loading of exogenous PSs, can improve their stability and therefore PDT [7]. Additionally, MOFs can be loaded with different therapeutics, such as immunotherapeutic agents (e.g., immune adjuvants and immunomodulators), for controlled delivery at the TME. As a result, MOFs have the potential to improve the treatment outcomes of PIT and synergetic photo-immunotherapy while addressing some of their limitations [118]. In this section, we will cover cancer therapy strategies that use MOFs as nanoplatforms for the combination of PDT and immunotherapy.

Immune checkpoints are essential proteins for the prevention of autoimmunity and the regulation of the amplitude and quality of T cell immune responses via stimulatory and inhibitory regulatory signals for T cell receptors (TCR) [150]. However, in tumor cells, this protein’s expression is significantly dysregulated, inhibiting antitumor immunity and allowing cancer cells to proliferate [150,151]. Immune checkpoint blockade (ICB) therapy emerged as a monoclonal antibody-based immunotherapy that aims to reduce immunosuppression by blocking immune checkpoints, consequently triggering enough immunostimulation to elicit an effective antitumor response [152,153]. Several antibodies have already been designed specifically targeting cytotoxic T lymphocyte antigen 4 (CTLA4) or programmed cell death 1 (PD1)-PD1 ligand 1 (PD-L1) [154]. PD-1 is a co-inhibitory receptor that binds to the ligands PD-L1 and PD-L2, which are expressed in both immune and non-immune cells and act as a “checkpoint” of T cell activation, playing an essential role in maintaining immunological homeostasis of the cell during infections [155]. These proteins are expressed by cancer cells as an adaptive resistance mechanism against immune cells, resulting in an immunosuppressive environment. As a result, the creation of anti-PD-L1/anti-PD-1 antibodies to inhibit these checkpoints can stimulate the activation of a stronger immune response [156]. However, cancer cells have inherent characteristics linked to genetic, transcriptional, and functional aspects that allow for mechanisms that bestow resistance against ICB, limiting the number of patients who react to the treatment [154]. Furthermore, because immunological checkpoints are present in both cancer cells and in normal cells, adverse effects associated with antibody therapy are very common, frequently severe, and persistent, restricting treatment administration [157].

**Figure 3:**
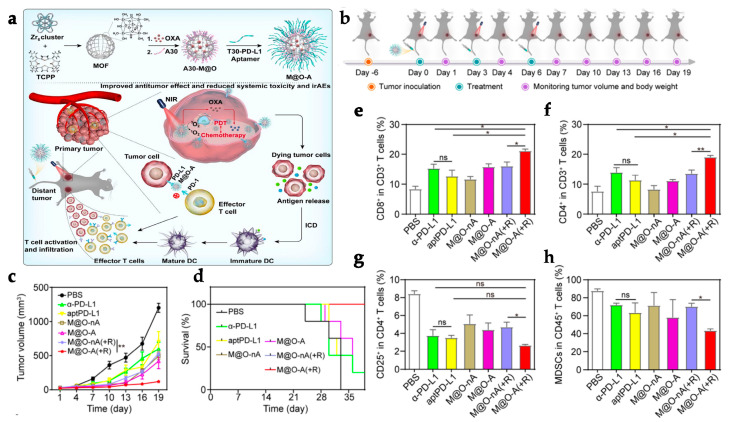
Representative example of an MOF used in synergetic PDT-immunotherapy. (**a**) M@A was synthesized by encapsulation of OXA in PCN-224 and subsequent adsorption of T30-PD-L1. M@A was designed for a combination of PDT, chemotherapy, and immunotherapy to improve ROS production and enhanced antitumor immune response. (**b**) MC38 tumor cells were inoculated in mice and grown to a tumor volume of 50–100 mm^3^. Mice were treated with 10 mg/kg MOF and irradiated or not with a laser at 640 nm (0.1 W/cm^2^) for 30 min at days 6, 9, and 12 after inoculation. (**c**) Tumor growth curves of mice injected with different treatments and with or without irradiation. (**d**) Survival rates of mice under different treatments with or without irradiation after >35 days. (**e**–**h**) Flow cytometry analysis of infiltrating (**e**) CD8^+^ T cells, (**f**) CD4^+^ T cells, (**g**) Treg cells and (**h**) MDSCs in the TME. *n* = 5. ns >0.05, * *p* < 0.05, ** *p* < 0.01. Reproduced with permission from [123] © 2022 Wiley-VCH GmbH.

To address the systemic immunotoxicity problem associated with the administration of immunotherapeutic antibodies such as α-PD-L1, Zhang et al. designed M@O-A (with M referring to MOF, O to oxaliplatin, and A to aptPD-L1) in a strategy that relied on the combined action of PDT, chemotherapy, and immunotherapy (Figure 3a). T30-PD-L1 aptamer or aptPD-L1 adsorption to the surface of PCN-224 NPs was validated by a dramatic shift in the zeta potential from 18.8 ± 0.5 mV to −26.1 ± 2.3 mV. The M@O-A composite was further synthesized by loading oxaliplatin (OXA) into PCN-224 MOF with a loading percentage of 15.9%. OXA was released as a result of a light-triggered mechanism. In a study to assess the controlled release of OXA, 100% of the content was released from PCN-224 under 1 min laser irradiation at 640 nm (0.1 m W/cm^2^). On the other hand, aptPD-L1 modification precisely targets and attach to tumor cells that express PD-L1. The application of the MOF resulted in an effect of enhanced permeability and retention (EPR) for effective accumulation and long-term retention of the M@O-A at the tumor site, whereas anchoring of aptPD-L1 to the MOF increased the stability of the aptamer and also extended the retention period at the tumor site, improving the immunotherapeutic effect. A 3 h incubation of 50 μg/mL of M@O-A NPs with colorectal MC38 tumor cells, followed by 15 min irradiation with an LED light at 640 nm (0.1 W/cm^2^), resulted in nearly 100% cell apoptosis because of the combined effect of PDT and chemotherapy. Furthermore, CRT overexpression confirmed that PDT/chemotherapy substantially triggered ICD. In vivo experiments were carried out on 60 MC38-Luc tumor-bearing mice (Figure 3b) with an average tumor volume of 50–100 mm^3^, that were randomly divided into twelve groups (*n* = 5 for each group). Mice were administered an M@O-A injection (10 mg/kg of body weight) every 3 days for a total of three times, followed by 30 min LED irradiation at 640 nm (0.1 W/cm^2^). The study reported an increase in cytokine levels as well as antitumor CD3^+^, CD3^+^CD4^+^ (Figure 3f), and CD3^+^CD8^+^ (Figure 3e) T cell proliferation while decreasing inhibitory immune CD25^+^CD4^+^ regulatory T cells (Tregs) (Figure 3g) and myeloid-derived suppressor cells (MDSCs) (Figure 3h), effectively changing the tumor microenvironment and inducing a strong antitumor immunity. Notably, M@O-A NPs + NIR light treatment dramatically inhibited primary tumor growth (Figure 3c), promoting a 100% survival rate (5/5) for more than 35 days (Figure 3d). In a similar procedure using a bilateral tumor model, aptPD-L1 treatment boosted systemic immune response, exerted abscopal effects, and completely inhibited distant tumor growth. Semi-quantitative histological examination of the colon, kidneys, liver, and spleen displayed that M@O-A treatment (equivalent aptPD-L1 dosage 100 nmol/kg, intravenously) with irradiation resulted in considerably lower histological scores (≤1) than those treated with α-PD-L1 (250 μg/mouse, intraperitoneally), implying significantly less systemic toxicity or immune-related adverse events (irAEs) [123].

In a different study, Ni et al. described the development of a novel Cu-porphyrin nanoscale MOF for enhanced ROS therapy in a combination of estradiol (E2)-induced chemotherapy and PDT for a more robust ICD and synergy with ICB for a systemic tumor inhibition [131]. E2 is a member of the steroid hormone family and acts by binding to the soluble intracellular receptors (ERα and Erβ) which proceed to the nucleus and carry a ligand-dependent transcription factor function [131,158]. Cell growth, cell cycle arrest, and carcinogenesis can all be directly impacted by receptors’ expression levels [131]. The 4-OH catechol of estradiol estrogen metabolite can generate ROS in reactions catalyzed by bioavailable Cu^2+^ ions for oxidative damage to DNA [159], making it a good target for effective radical therapy. Cu-TBP (with TBP referring to tetrabenzoatoporphyrin) was generated by sonicating a mixture of CuCl_2_ and 5,10,15,20-tetrabenzoatoporphyrin (H_4_TBP). Cu-TBP was presumed to be only metastable inside cells since, under acidic pH conditions (5.5 and 4.5), mimicking the lysosome, the nanoplates proceeded to break down up to 50 and 75%, respectively, enabling the release of Cu^2+^ and TBP ligands inside tumor cells. The dual triggered radical therapy was evaluated using cancer cell lines with high (human ovarian cancer cell SKOV-3 = 140.35 ± 13.45 pg/10^6^ cells and murine melanoma cell B16F10 = 124.25 ± 8.78 pg/10^6^), medium (human prostate cancer cell PC-3 = 53.80 ± 9.23 pg/10^6^), and low (human colorectal cancer HCT-116 = 8.30 ± 4.36 pg/10^6^) concentrations of E2. Treatment with Cu-TBP (0–100 μM of TBP concentration ) and LED light (650 nm at 0.1 W/cm^2^) for 15 min confirmed the synergy between PDT and Cu-E2 redox cycle as light treatment decreased the IC50 values from 25.68 ± 5.67, 41.33 ± 8.87, 57.23 ± 10.12 and >100 mM in the dark to 4.57 ± 2.45, 6.37 ± 4.26, 19.73 ± 6.78, and 34.52 ± 7.23 mM for SKOV-3, B16F10, PC-3, and HCT116 cells, respectively. The enhanced ROS production driven by the combination of PDT and E2-triggered chemotherapy had a strong cytotoxic impact, triggering apoptosis in 79.1% of the cells in B16F10 cells treated with 20 μM (concentration of TBP) Cu-TBP when exposed to an LED light (650 nm, 0.1 W/cm^2^) for 15 min. Due to increased ROS production, Cu-TBP and light treatment could also cause significant DNA double-strand breaks (DSB) and lipid peroxidation in the cells. In mice injected with tumor B16F10 and SKOV-3 cells, treatment with 0.2 μmol Cu-TBP exposed to LED light at 650 nm (0.1 W/cm^2^) for 30 min significantly suppressed tumor growth reaching tumor growth inhibition indices (TGIs) of 96.6% in B16F10 and eradication (100% in the 6 mice) in SKOV-3 cells. Higher ROS levels also resulted in a more robust ICD induction and phagocytosis by DCs, leading to improved antigen presentation and immune activation. Combination therapy with α-PD-L1 (75 μg per mouse) therapy resulted in increased infiltration of CD45^+^ (11.72% ± 5.41% and 8.84% ± 2.84% vs. 3.53% ± 1.25% and 1.53% ± 0.73% in PBS), CD4^+^ (1.60% ± 0.81% and 0.81% ± 0.17% vs. 0.29% ± 0.15% and 0.20% ± 0.17% in PBS) and CD8^+^ (2.62% ± 2.35% and 0.54% ± 0.26% vs. 0.14% ± 0.14% and 0.04% ± 0.03% in PBS) T cells in primary and distant tumors, respectively. The cumulative effects of a systemic immune response culminated in a significant tumor-specific T cell activation, successfully suppressing local (98.3% TGI) and distant (94.4% TGI) tumors, completely curing two of the six mice treated (33.3% cure rate), and extending the median survival time from 23.5 (Cu-TBP and light treatment) to 31 days in combination treatment with α-PD-L1 [131]. This study leveraged the MOF’s versatility to widen the therapeutic impact of ICB employing hormonal therapy, inspiring the implementation of similar techniques in hormonally dysregulated tumors.

Xie et al. created a π-extended Pd-TBP doped porphyrin nMOF (PTP) that can measure radiometric O_2_ concentration and enhance PDT performance in cancer treatment [130]. In a series of reactions, PdCl_2_ was coordinated with TCPP, coupled with a Zr cluster as ligands (PTP) (zeta potential = 23 mV), and was further modified with a 4T1 membrane coating to form PTP@M (zeta potential = −24.7 mV) [130]. Tumor cell membranes have been shown to express surface antigens with homophilic adhesion domains, responsible for intercellular adhesion, endowing them with innate homotypic targeting capabilities towards cancer cells of the same kind [160,161]. The π-extended Pd-TBP induced a red-shifting effect on the PTP Q bands (589 and 630 nm), resulting in increased light usage efficiency and ^1^O_2_ generation. Under 630 nm (0.03 W/cm^2^) light irradiation for 5 min, 5 5 μg/mL of PTP induced higher ^1^O_2_ yield (28.5-fold fluorescence enhancement of singlet oxygen sensor green (SOSG)) than a porphyrinic MOF (PMOF) (8.1-fold). Furthermore, PTP could induce higher ^1^O_2_ production in 10% O_2_ (20.2-folds) and 1% O_2_ (10.9-folds) hypoxic environments. Oxygen levels were shown to affect ROS production and therefore the cytotoxicity of PTP. PTP (30 μg/mL) under irradiation (630 nm at 0.03 W/cm^2^ for 5 min) was shown to trigger apoptosis in 73.1%, 55.2%, and 15.2% of cells in 20%, 10%, and 1% O_2_ environments, respectively. PTP@M was shown not only to be an excellent platform PDT with improved ROS generation owing to the doping of π-extended Pd-TBP but also for diagnostics due to tumor cell homotypic targeting and long-term residency. In vivo studies showed that, in a 4T1 tumor model, mice injected with PTP@M (200 μL, 1 mg/mL) and subjected to a 630 nm (0.3 W/cm^2^) laser for 5 min inhibited cancer development due to increased PDT cell killing, culminating in a robust ICD and immune response activation. Furthermore, combining PTP@M and light (630 nm, 0.2 W/cm^2^, 5 min) treatment with the checkpoint inhibitor PD-1 (4 mg/kg) boosted tumor-infiltrating CD8^+^ T cell proliferation, resulting in greater tumor suppression and anti-metastasis effects. The lack of damaged tissues in major organs shown by H and E staining indicated that PTP@M was not toxic in normal tissues [130].

In an alternative CBI strategy, Lu et al. described a therapeutic approach that combines PDT and immunotherapy by encapsulating indoleamine 2,3-dioxygenase inhibitor (IDOi) in a chlorin-based nanoscale MOF (TBC-Hf, with TBC, referring to 5,10,15,20-tetra(p-benzoato)chlorin (H_4_TBC)), thereby producing IDOi@TBC-Hf, to elicit a systemic immune response [136]. Indolamine 2, 3-dioxygenase (IDO) is an immune checkpoint that catalyzes the first and rate-limiting step of tryptophan (Trp) catabolism to kynurenine, suppressing T cell proliferation and inducing T cell differentiation and apoptosis. IDO is markedly overexpressed in cancer; it has an immunosuppressive effect in the antitumor immune response [136,162]. IDOi was loaded into the TBC-Hf to a loading weight percentage of 4.7%. When incubated in Hank’s balanced salt solution (HBSS) for 24 h, IDOi@TBC-Hf released 83.3% of IDOi content. Compared to TBP-Hf, containing the porphyrin ligand TBP, TBC-Hf absorbs more effectively red light. TBP-Hf presents a Soret band at λ_max_ = 418 and Q bands at 517, 550, 593, and 647 nm, while TBC-Hf is at λ_max_ = 421 and Q bands slightly red-shifted to 520, 548, 600, and 653 nm, thereby increasing the efficiency of absorption. This difference enhanced the ^1^O_2_ production and PDT efficacy of TBC-Hf, acting as a more efficient PS. In vitro studies proved the efficiency of PDT using CT26 and MC38 cells incubated with 1 μM (TBC equivalent concentration of 2 μM) TBC-Hf and irradiated with a LED light (650 nm, 0.1 W/ cm^2^) for 15 min; these studies demonstrated a higher rate of necrosis and apoptosis (70% in CT26 and 39.48% in MC38) compared to TBP-Hf (44.4% in CT26 and 11.38% in MC38). Furthermore, cells treated with both TBC-Hf and TBP-Hf exhibited higher expression of CRT, a sign of ICD induction. The strategy relies on the fact that the PDT-induced ICD would synergize with the release of IDOi at the local TME and blood circulation for systemic IDO blockage and immune activation. The authors demonstrated that, in a bilateral mouse model of CT26 and MC38 cancer cells, treatment with 20 μmol/kg of IDOi@TBC-Hf and LED light at 650 nm (0.1 W/cm^2^) for 15 min led to the near elimination of primary tumors, reducing tumor sizes to 1.1 ± 0.2% and 0.8 ± 0.3% of the PBS-treated control in CT26 and MC38 cells, respectively. Furthermore, the treatment also sorted abscopal effects with a reduction of distant tumor sizes 6 and 5 days after treatment in CT26 and MC38, respectively. As a result of the synergy of PDT-induced ICD and IDOi immunotherapy, a systemic antitumor immune response was induced for an effective primary and distant tumor rejection. After 14 days, an ELISPOT assay in MC38 models revealed an increase of infiltrating neutrophils (*p* = 0.0369 vs. PBS) and B cells (*p* = 0.0215 vs. PBS) at primary and distant tumors 12 h after treatment. 12 days after treatment, the infiltration of CD4^+^ T cells (*p* = 0.0206 vs. PBS in and *p* = 0.0388 vs. PBS) increased for primary and distant tumors, respectively, and CD8^+^ T cells (*p* = 0.0012 vs. PBS) and NK cells (*p* = 0.0034 vs. PBS) in distant tumors. Therefore, this work described a synergetic strategy with the potential to enhance systemic tumor-specific immunotherapy in cancer treatment, using a MOF nanoplatform [136].

Bai et al. focused on the application of an MOF photosensitive nanointerferer to increase tumor cells intrinsic immunogenicity and mobilize the immune system to identify and eradicate tumors by inhibiting Cyclin-dependent kinase 4 (Cdk4) and activating PDT to promote immunogenic tumor antigen production and presentation. The development of msiPCN began with the condensation of a small interfering RNA (siCdk4) to knock down Cdk4 and cationic protamine for protection against enzymatic degradation and facilitated lysosome escape through a “proton sponge” effect. The protamine-encapsulated siCdk4 was further linked and loaded into PCN-224 with a 77% loading efficiency. The generated siPCN was coated with murine colon carcinoma cells (CT26) cell membranes, which drastically reduced the zeta potential from positive to negative. Hence, the authors proved that the CT26 tumor cell membrane coating enhanced selective targeting of msiPCN in CT26 cancer cells, rather than other cell types via accumulation and receptor-mediated specific endocytosis. When cellular uptake profiles of CT26 cells and murine breast tumor cells (4T1) were compared, msiPCN (7.5 μg/mL) entered CT26 cells within 2 h and continued to increase until 6 h, resulting in 6.6-fold higher endocytosis than 4T1 cells. Light irradiation at 660 nm (0.03 W/cm^2^) for 15 min on CT26 cells incubated with 7.5 μg/mL msiPCN resulted in significant cytotoxicity to tumor cells, with cell viability below 50%. However, the findings highlighted the need for PDT to coordinate with siCdk4 to achieve greater results. Cell cycle progression was also hindered by msiPCN downregulation of Cdk4; this was most prominent in the G0/G1 and S phases (69.43% and 12.05%, respectively) and successfully prevented cell division and proliferation. Furthermore, siCdk4 displayed direct immunomodulatory effects, increasing the levels of PD-L1 protein and the expression of major histocompatibility complex (MHC) class I, which is important in antigen presentation. The siCdk4 further synergizes with PDT for a stronger ICD, increasing tumor cell immunogenicity and mobilizing a powerful immune response. 35 CT26 tumor-bearing mice were randomly divided into five groups (*n* = 7) and treated every 4 days with intravenous administration of 1 mg/mL of the materials under study, before being irradiated with a He-Ne laser at 660 nm (0.15 W/cm^2^) at the tumor site for 2 min. The treatment with irradiation msiPCN demonstrated that synergetic therapy was beneficial in slowing tumor development when compared to the control group, with more than 30% of the mice surviving 30 days. When combined with anti-PD-L1 antibodies administration (75 μg per mouse, subcutaneously), therapeutic effects were magnified. PDT-induced ICD, cell cycle arrest, and increased PD-L1 proteins improved antitumor immunity by activating important immunological effector cells such as CD8^+^ T cells; they were consistently the best treatment group at tumor growth suppression, reaching 100% mice survival rate after 30 days. After 30 days, hematoxylin and eosin (H and E) staining of key organs revealed no significant pathological changes in groups treated with msiPCN nanocomposite. As a result, this study provided an alternative synergistic way to boost tumor photoimmunotherapy in conjunction with Cdk4 inhibition, which could effectively reduce tumor growth with negligible toxicity [124].

DCs are essential for immune activation because they present antitumor antigens to T cells, triggering an antitumor immune response [163]. Toll-Like Receptor (TLR) 9 is a pattern recognition receptor that activates protective adaptive immunity in response to intracellular pathogen infections by recognizing specific conserved structures [164]. Immune adjuvants are immune enhancers that stimulate immune cell activation for the induction of immune responses. Unmethylated cytosine-phosphate-guanine (CpG) are synthetic oligodeoxynucleotides (CpG-ODN) composed of a single strand of synthetic DNA with a sequence of cytosine triphosphate deoxynucleotides (C) linked to guanine triphosphate deoxynucleotides (G) through phosphodiester bonds. CpG sequence repeat is common in bacterial and other prokaryotes DNA [165]. Therefore, CpG is a well-known adjuvant, primarily detected by TLR9, that stimulates several immune cell subsets (T cells, B cells, NK cells, DCs, monocytes, and macrophages) to promote an immunological response [166]. However, the efficacy of free CpG is significantly hampered by its anionic surface, which renders the penetration of cell membranes into the intracellular microenvironment a challenge. Moreover, in physiological conditions, CpG is prone to degradation by nucleases [167].

To solve enzymatic degradation and ineffective cellular internalization issues of anionic CpG oligodeoxynucleotide for DC activation in vivo, Ni et al. created a W-based MOF for efficient PDT and CpG delivery. To generate the composite W-TBP/CpG (W standing for tungsten and TBP to 5,10,15,20-tetra(p-benzoato)porphyrin), CpG was adsorbed to the surface of the cationic rectangular nanoplate-like W-TBP with an efficiency of 87.9%. When compared to free CpG in a 72-h incubation of DCs harvested and differentiated from bone marrow cells, CpG adsorption to MOF had a favorable effect on delivery to DC, exhibiting elevated levels of the cytokines IFN- and IL-6 (DC maturation markers). To assess W-TBP cytotoxicity, BALB/c mouse mammary cancer cells (TUBO) were cultured for 8 h with various concentrations (0–100 M) of the different study groups before being irradiated with light at 650 nm (0.1 W/cm^2^) for 7.5 min. At a maximum concentration of 100 μM, cells treated with irradiated W-TBP exhibited approximately 80% apoptotic cell death. Furthermore, cells incubated with W-TBP (at an equivalent TBP concentration of 20 μM) and exposed to light demonstrated significant amounts of ROS generation and CRT exposure, both of which are hallmarks of PDT-induced ICD. In vivo experiments in a TUBO-tumor bearing murine breast adenocarcinoma model of five mice treated with W-TBP/CpG (at a TBP dose of 0.2 mol and a CpG dose of 1 g administered intratumorally) without irradiation resulted in improved tumor regression at day 22, indicating the ability of the nanocomposite to deliver CpG to DCs in the TME. PDT (W-TBP) alone displayed insufficient antitumor efficacy when irradiated with light at 650 nm (0.1 W/cm^2^) for 7.5 min, as opposed to W-TBP/CpG, which resulted in 96.6% tumor regression due to the synergistic effect of PDT and CpG delivery. W-TBP/CpG-irradiated cells had elevated MHC-II and costimulatory CD86 molecules (66.9%); this was compatible with the increased CpG-induced DC maturation (64.0%) and ICD-mediated antigen presentation. While W-TBP/CpG and irradiation alone had essentially little effect on distant tumors in a bilateral model of TUBO tumors on BALB/c mice, when paired with α-PD-L1 (75 mg/mouse), this synergistic treatment showed substantial abscopal effects, with more than 97% tumor regression in both local and distant tumors. Irradiation combined with W-TBP/CpG/α-PD-L1 treatment boosted leukocyte and CD4^+^ and CD8^+^ T cell infiltration in both local and distant tumors [128]. As a result, due to employing a photosensitizing MOF, this work proposes a novel strategy for antigen presentation and immune activation for cancer photoimmunotherapy.

In most malignant tumors, it is common for hypoxia to occur, a phenomenon that overdevelops the tumor by a non-physiological level of oxygen tension (outstrips of oxygen supply) [168,169]. Notably, numerous mechanisms, including partway hypoxia-inducible factor-1 (HIF-1) in combination with hypoxia, influence the majority of cancer hallmarks (cellular proliferation, apoptosis, metabolism, immunological responses, genomic instability, vascularization, neovascularization, invasion, and metastasis) [168]. HIF-1 is a heterodimer comprised of a HIF-1α subunit and a constitutive HIF-1β subunit. HIF-1α is an oxygen-regulated protein. Under normoxic circumstances, the protein has an extremely short half-life as it is constantly synthesized and degraded. Conversely, in hypoxic circumstances, HIF-1α is not degraded and constantly accumulates protein as a result of enhancing protein transcription across several pathways, including the expression of immunosuppressive molecules [170,171,172,173]. In addition, hypoxic TME compromises PDT efficiency due to PSs oxygen requirement to produce ROS [170].

Lan et al. created Fe-TBP using MOF structures to boost PDT efficiency as well as PD-L1 ICB by overcoming TME hypoxia and promoting immunotherapeutic effects. Fe-TBP was created by combining Fe_3_O clusters and the ligand 5,10,15,20 tetra(p-benzoate)porphyrin (TBP) in a ratio of 2.21. The higher Fe to TBP ratio was most likely caused by the nanosize or a defect in the Fe-TBP nanocomposite. Under hypoxic settings, cancer cells generally contain high quantities of H_2_O_2_. When exposed to such conditions, Fe-TBP undergoes a Fenton reaction to create O_2_, which is then transformed into singlet oxygen (^1^O_2_) by the excited porphyrin ligands. By incubating 150 μM of H_2_O_2_ with 50 μM of Fe-TBP in oxygen-free phosphate buffer saline (PBS) solution, the catalytic activity of Fe-TBP for O_2_ production was evaluated. Fe-TBP was able to produce significant amounts of oxygen (>1.5 ppm) after 50 min. The authors further demonstrated the MOF’s ability to overcome hypoxia by assessing the protein expression of HIF-1 α by immunostaining CT26 cells in vitro and CT26 tumor-bearing mice in vivo through treatment under hypoxic and normoxic conditions. Under a hypoxic environment, the intensity of HIF-1 fluorescence dropped considerably when treated with Fe-TBP (at an equivalent ligand dosage of 10 μM in vitro and 0.2 μmol in vivo), demonstrating hypoxia relief at the tumor level. Notably, 81.2% of CT26 cells treated with Fe-TBP at an equivalent ligand dose of 10 μM and LED light at 650 nm (0.1 W/cm^2^) for 15 min underwent an apoptosis state. As evidenced by a higher CRT expression in treated local tumors, Fe-TBP could further mediate effective PDT-induced ICD under normoxic and hypoxic conditions. In vivo studies in a bilateral CT26 tumor-bearing murine model demonstrated that treatment with Fe-TBP at a TBP dosage of 0.2 μmol irradiated with an LED light at 650 nm (0.1 W/cm^2^) for 7.5 min almost completely inhibited primary tumor growth. Even so, Fe-TBP-PDT treatment had a minor influence on distant tumors. In contrast, in combination with α-PD-L1 (75 mg/mouse), the immunotherapeutic impact of Fe-TBP was significantly enhanced, inducing more than 90% tumor regression in local and distant tumors and increasing tumor-specific T cells such as infiltrating CD4^+^ and CD8^+^ T cells. As a result, Fe-TBP is proposed as a new nanoplatform capable of both overcoming TME hypoxia for a more efficient PDT and combining PDT and ICB to induce systemic antitumor immunity [129].

**Figure 4:**
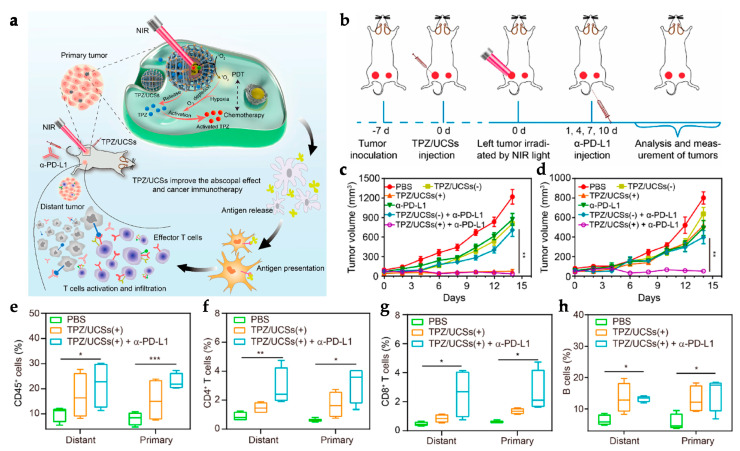
MOFs used for synergetic PDT-immunotherapy (**a**) TPZ/UCS promoted UCNP core up conversion luminescence to Zr-MOF shell enhancing PDT. TPZ encapsulated in Zr-MOF nanopores is activated by hypoxia synergizing with PDT for ICD induction. Antigen release activates immune response. The combination with α-PD-L1 further increased antitumor immune response. (**b**) CT26 tumor cells inoculated in a bilateral tumor model in mice (grown to 50–100 mm^3^). Mice were treated with UCS/TPZ (25 mg/kg) and UCS laser irradiated (980 nm, 1.2 W/cm^2^) for 20 min (5 min break after 1 min irradiation). 750 μg/kg α-PD-L1 was injected on days 1, 4, 7, and 10 after treatment. (**c**,**d**) Tumor growth curves of the primary (**c**) and distant (**d**) tumors in different treatment mice. (**e**–**h**) Percentage of infiltrating CD45^+^ cells, (**e**) CD4^+^ T cells, (**f**) CD8^+^ T cells (**g**), and B cells (**h**) in total tumor cells in primary and distant tumors. Data are means ± SD. * *p* < 0.05, ** *p* < 0.01, and *** *p* < 0.001. Adapted with permission from [135]. Copyright © 2020 American Chemical Society.

Shao et al. developed a distinct strategy, designing a core-shell upconversion nanoparticle@porphyrinic MOFs (UCSs) as a synergistic treatment combining PDT, chemotherapy, and immunotherapy against hypoxic tumors (Figure 4a). The TPZ/UCS (TPZ refers to tirapazamine) composite was composed by a core of lanthanide-doped upconversion nanoparticles (UCNPs) and a shell of porphyritic MOF assembling an heterostructure that favors higher energy transfer efficiency from the UCNP core to the MOF for an enhanced singlet oxygen (^1^O_2_) generation. UCNPs were modified with a citrate acid (CA) coating (zeta potential reduction to −4.7 mv) to mediate the growth of the MOF at the surface. Through a heterogenous nucleation process regulated by the presence of CA, a porphyrinic MOF (Zr_6_ cluster + TCPP) shell structure grew at the surface, originating the heterostructure of the UCS. Under light irradiation (980 nm), the upconversion luminescence (UCL) of the UCNPs exhibited three peaks of Er^3+^ centered with a good overlap with the absorption spectrum of the porphyrinic MOFs, making it perfect for an efficient resonance-based energy transfer (FRET). UCSs spectra displayed significantly lower intensity peaks, indicating an efficient FRET from the UCNPs to the MOF within the UCSs. TPZ, a hypoxia-activatable prodrug, was then encapsulated into the nanopores of the MOF shell with a 10 wt% efficiency for synergistic PDT and chemotherapeutic treatment. Under acidic conditions (pH 5.5), TPZ/UCS (1 mg/mL) displayed a release rate of TPZ of around 80%. In vitro assays performed under hypoxic (2% oxygen levels) and normoxic (21% oxygen levels) conditions to assess the cytotoxicity of nanoparticles on CT26 cells revealed that TPZ/UCSs without irradiation had half-maximal inhibitory concentrations (IC_50_) of 3.02 and 55.04 μg/mL and cell viability of less than 50% and 100%, respectively. In contrast, under 980 nm (1.2 W/cm^2^) irradiation with a 3 min break for every minute of irradiation, TPZ/UCSs demonstrated increased cytotoxicity under hypoxic settings with an IC_50_ of 0.74 μg/mL; cell viability reduced to around 25%. Thus, the combined therapy of NIR light-induced PDT and hypoxia-triggered chemotherapy increased the lethal impact of TPZ/UCS on tumor cells through the production of ROS. Treatment with TPZ/UCS plus irradiation at 980 nm (1.2 W/cm^2^) for 20 min (with a 5 min pause for every minute of irradiation) significantly inhibited tumor growth in mice injected with CT26 cells. When compared to the other study groups, H and E staining of sections of tumors excised from mice treated with TPZ/UCS plus irradiation revealed significant tumor tissue necrosis, lowering the density of living tumor cells by 28.9%. Furthermore, the higher expression of CRT indicated a strong ICD induction. Synergy with PD-L1 inhibition therapy (750 μg/kg PD-L1 antibody injected intravenously once every 3 days) in a bilateral CT26 tumor model (Figure 4b) successfully raised the number of infiltrating CD45^+^ (22.84 ± 2.97% and 21.74 ± 8.32%, respectively), CD4^+^ (3.15 ± 1.14% and 2.88 ± 1.45%, respectively), CD8^+^ T (2.60 ± 1.29% and 2.58 ± 1.75%, respectively) cells, and NK cells (3.05 ± 1.11% and 2.19 ± 0.95%, respectively) (Figure 4e–h). As a result, the combination of irradiated TPZ/UCSs and α-PD-L1 effectively suppressed the development of both primary and untreated distant tumors (Figure 4c,d), resulting in consistent systemic antitumoral effects [135]. This work established a potentially useful nanoplatform for treating hypoxic tumors using a combination of PDT, chemotherapy, and immunotherapy.

In a recent study, a novel MOF system that can be employed as an in situ tumor vaccine to counteract cancer hypoxia signaling, can improve PDT efficiency, and promote long-term antitumor immunity has been developed, showing promising outcomes [127]. The nanoparticles PCN-ACF-CpG@HA were synthesized by encapsulating acriflavine (ACF) (8.3 wt%) followed by the adsorption of the immune adjuvant CpG (1.45 wt%) and HA (45.35 wt%) to the MOF surface, causing a decrease of the zeta potential from 2.85 mv to −20.27 mV [127]. ACF is a drug that prevents HIF-1 α dimerization, which inhibits HIF-1 α DNA binding and subsequent transcriptional activity, resulting in tumor growth inhibition, circulating angiogenic cells (CACs) mobilization, and tumor vascularization [127,174]. The HA coating enables specific targeting and improved cellular absorption at the tumor site, as well as HAase-mediated release of CpG and ACF in the TME. The release behaviors of ACF and CpG in 4 mg/mL PCN-ACF-CpG@HA were evaluated using a PBS dialysis system under laser irradiation (670 nm, 0.1 W/cm^2^, 5 min) as well as the addition of HAase (5 mg/mL). Irradiation and the addition of HAase increased the release of ACF and CpG from 21% to 63% and 12% to 44%, respectively. Authors further demonstrated, through the use of immunofluorescent staining of murine hepatic carcinoma cells (H22) treated with PCN-ACF-CpG@HA under light irradiation (670 nm, 0.1 W/cm^2^, 5 min), that ACF inhibits overexpression of survival/metastasis linked genes regulated by HIF-1α rather than inhibiting HIF-1α since it only blocks dimerization without affecting the expression. Hence, after PDT treatment, PCN-ACF-CpG@HA could significantly block HIF-1α -mediated cell survival and metastatic signaling. In vitro studies revealed that the synergistic impact of PDT (670 nm, 0.1 W/cm^2^, 5 min) and anti-hypoxic signaling in H22 cells treated with 32 g/mL (final PCN concentration) of PCN-ACF-CpG@HA promoted higher cytotoxic effects, resulting in severely low cell viability (11%). Moreover, the release of immune adjuvant CpG in combination with PDT-induced ICD could promote stronger DC maturation (percentage of CD11c^+^, CD86^+^, and CD80^+^ = 70.68%); this is consistent with higher percentages of CD11c^+^/MHCII^+^ cells (57.3%), CD11c^+^/CD317^+^ cells (67.8%), and higher cytokine secretion. Irradiated (with a laser at 670 nm at 0.25 W/cm^2^ for 10 min) PCN-ACF-CpG@HA (10 mg/kg) treatment of H22-bearing mice demonstrated inhibition of hypoxia-induced cell survival and metastasis signaling genes, persistent high DC maturation (61.21%), and subsequent increase in CD8^+^ T cell and CD4^+^ T cell infiltration at the tumor site, resulting in efficient tumor suppression and metastasis prevention. The nanoplatform did not exhibit any evidence of systemic toxicity either; this was determined by biochemical analyses carried out 16 days after injection, which revealed no abnormal indexes. Likewise, H and E staining revealed no evidence of organ damage [127]. This unique MOF system is described as promising for the development of synergistic cancer therapeutic approaches using PDT.

Autophagy is a tightly controlled process that manages cellular damage resulting from environmental or genetic factors, as well as nutrient deprivation and aging. The various processes culminate in the degradation of the damaged intracellular components by lysosomes [175]. Mitophagy, for instance, is characterized as cargo-specific autophagy in which damaged mitochondria are selectively removed via engulfment into vesicles coated with the ubiquitin-like protein MAP1 light chain 3 (LC3) to aid in the growth and sculp of the isolation membrane and cargo recruitment. Once mitochondrial depolarization occurs, Parkin, an E3 ubiquitin ligase, is recruited and translocates from the cytosol to the mitochondria to mediate mitochondrial ubiquitination [176]. Because both mitophagy and apoptosis are initiated on the outer mitochondrial membrane, mitophagy can be either pro-death or pro-life. The cell fate is determined by the engulfment of a single mitochondrion in a pro-living role or the self-commitment to apoptosis in circumstances with significant mitochondrial damage, releasing cytochrome C for additional damage in the mitochondria via ROS production [177].

**Figure 5:**
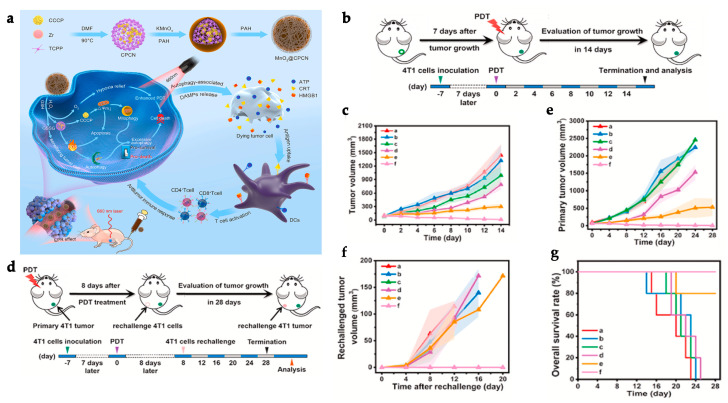
MOF-based synergetic PDT-immunotherapy (**a**) PCN-224 encapsulated CCCP through a solvothermal reaction and was coated by a MnO_2_ shell in a redox reaction between KMnO_4_ and PAH. A coating of PAH was added generating MnO_2_@CPCN. Synergy of PDT with autophagy/mitophagy pro-death function and its immunomodulating effects triggered increased immune response. (**b**) 4T1 tumor cells were inoculated in mice and grown for 7 days. Mice were treated with (**a**), PBS; (**b**), Laser; (**c**), MnO_2_@CPCN (12 mg/kg PCN equiv. and 3 mg/kg CCCP equiv.); (**d**), MnO_2_@PCN + L (12 mg/kg PCN equiv.); (**e**), CPCN + L (3 mg/kg CCCP equiv.); and (**f**), MnO_2_@CPCN + L. (12 mg/kg PCN equiv. and 3 mg/kg CCCP equiv.) and laser irradiated (660 nm, 0.2 W/cm^2^) for 10 min. Tumor evolution was evaluated for 14 days until mice were sacrificed. (**c**) Tumor volume under different treatments. (**d**) Mice were inoculated with 4T1 tumor cells and treated after 8 days. Tumor growth was evaluated for 28 days. (**e**,**f**) Tumor volume variation of primary (**e**) and rechallenged tumor (**f**) after treatment. (**g**) Survival rates after different treatments. *n*= 5 [125]. Copyright © 2022, with permission from Elsevier.

A recent study by Sun et al. described the design of a MOF-based nanoplatform to enhance PDT therapy by taking advantage of the autophagy/mitophagy pro-death function and its immunomodulating effects. To induce self-protective mitophagy, the mitochondrial uncoupler carbonyl cyanide 3-chlorophenyl-hydrazone (CCCP) was solvothermally encapsulated in the porous porphyrinic PCN-224 with a loading efficiency of 95.7%, yielding CPCN (Figure 5a). A redox reaction between polyallylamine hydrochloride (PAH) and KMnO_4_ led to the formation of a MnO_2_ shell on the surface of CPCN. To increase biocompatibility and solubility, an electrostatic tethering of PAH was added, resulting in the final nanoplatform, MnO_2_@CPCN (Figure 5a). The deposition of MnO_2_ and the tethered cationic polyelectrolyte to the surface of the nanocomposite resulted in the increase of the zeta potential to 39.3 ± 4.8 mV. As a glutathione scavenger and “gatekeeper” for CCCP delivery, the MnO_2_ shell was presented as an essential part of this nanoplataform, preventing the premature release of CCCP. Contact with glutathione would cause the MnO_2_ shell to decompose and thus the CCCP to be released, leading to mitochondrial depolarization. Upon incubation in a GSH-free PBS solution, MnO_2_@CPCN released little to no CCCP. In contrast, when incubated with different concentrations of GSH (0,5 and 10 mM), MnO_2_@CPCN released substantial quantities of CCCP after only a 4 h incubation. Additionally, MnO_2_ could catalyze the conversion of H_2_O_2_ to O_2_, relieving tumor hypoxia and improving PDT efficiency. When 4T1 cells were treated in vitro with MnO_2_@CPCN (8 μg/mL PCN equivalent and 2 μg/mL CCCP equivalent concentration) and 5 min of laser irradiation at 660 nm (0.03 W/ cm^2^), CCCP mitochondrial depolarization combined with PDT damage has proven to trigger higher rates of autophagy/mitophagy (0.36 Pearson’s correlation potential in comparison to 0.19 of the control group (PBS)), inducing autophagic cell death and therefore improving the cytotoxic effect in tumor cells (93% apoptosis proportion). Moreover, excessive autophagy proved to further activate ICD and DAMPs release. Higher HMGB1 (2.2-fold) release, ATP secretion (9.3-fold), and higher CRT expression were reported in comparison to the control group. Accordingly, 4T1 tumor-bearing mice, injected intravenously with MnO_2_@CPCN (12 mg/kg PCN equivalent and 3 mg/kg CCCP equivalent) and exposed to light irradiation at 660 nm (0.2 W/cm^2^) for 10 min, managed to inhibit tumor growth (Figure 5b), leading to eradication of tumor tissues in about 20% (1/5) of treated mice (Figure 5c). MnO_2_@CPCN + L treatment has also been shown to increase autophagy/mitophagy levels, upregulating the ubiquitin proteins LC3 and Perkin. Excessive pro-death mitophagy and PDT combined to powerfully induce ICD, subsequently triggering a robust antitumor immune response. Compared to the PBS control group, MnO_2_@CPCN plus light treatment increased by 7.7-fold the recruitment of mature DCs (34.7%) and by 5.1-fold and 4.4-fold the infiltration of CD4^+^ and CD8^+^ T cells in the tumor tissue, respectively. During the 28-day study (Figure 5d), mice treated with irradiated MnO_2_@CPCN showed clear primary tumor regression (Figure 5e) with no indication of recurrence after a rechallenging study (Figure 5f), yielding a 100% (Figure 5g) survival rate. The increase in the population of memory CD4^+^ T cells (39.4%) and memory CD8^+^ T cells (44.7%) in the spleen tissues of the mice after 28 days suggested antitumoral immunological memory that prevented tumor metastasis and recurrence [125]. This work demonstrated the nanoplatforms adaptability as nanocarriers for the application of various synergistic strategies that may offer a more straightforward way to treat solid tumors.

Until now, PDT has only been used to treat superficial tumors such as skin, neck, and oral cavity cancers. PDT’s capacity to treat deep tumors is severely restricted by the low tissue penetration depth of excitation light. The NIR region, featuring 650 to 900 nm wavelengths, achieves the best deep tissue penetration. Photon scattering and absorption by tissue (proteins, nucleic acids, hemoglobin, and melanin) restrict penetrating depth at wavelengths below 650 nm. On the other hand, water molecules may absorb photons at wavelengths above 900 nm. Most PSs available for clinical use absorb at a relatively low wavelength in the NIR “window,” directly implicating the efficiency of PDT in deep tumors [178].

**Figure 6:**
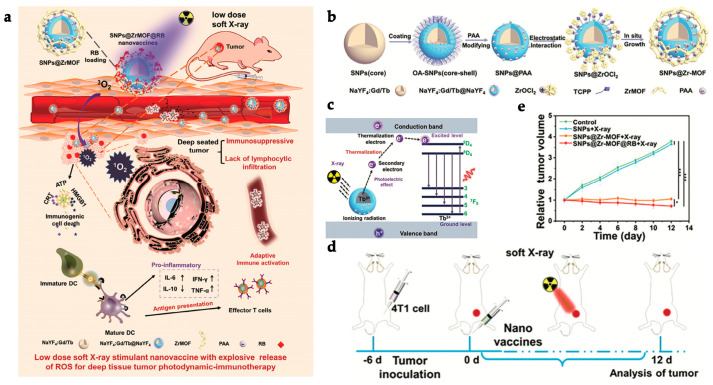
MOF-based synergetic PDT-immunotherapy strategy. (**a**) Combination of radiotherapy with PDT in SNPs@Zr-MOF@RB enhanced ROS production to promote a more efficient PDT in deep tumors. Robust PDT-induced ICD, led to an antitumor immune response and effective tumor cell killing. (**b**) Designing of SNPs@Zr-MOF@RB: NaYF4:Gd,Tb@NaYF4 SNP core coated by oleic acid (OA) is modified by the negatively charged polyacrylic acid (PAA) to promote the growth of a Zr-MOF at the surface through electrostatic interactions. (**c**) The photoelectric effect and Compton scattering, triggered by soft X-ray photons, generate free electrons and holes in the inner core of heavy atoms (Tb^3+^). Thermalization of free electrons and holes into the valence and conduction bands results in the emission of XEL from the excited triplet state through a radiative transition. (**d**) 4T1 tumor cells were injected in mice and grown for 6 days. Mice were injected every 3 days and exposed to X-rays every day at a tube voltage of 10–50 kV for 5 min. Different conditions tested were (1) PBS control, (2) SNPs (3 mg/mL) + X-ray, (3) SNPs@Zr-MOF (3 mg/mL) + X-ray, and (4) SNPs@Zr-MOF@RB (3 mg/mL) + X-ray. (**e**) Relative tumor volume for different treatment groups. Reproduced with permission from [138] © 2021 Wiley-VCH GmbH. * *p* < 0.05, *** *p* < 0.001.

Zhao et al. designed a synergy strategy for deep tissue PDT in combination with antitumor immunity using a soft-X-ray stimulated nanoprobe SNPs@Zr-MOF@RB (SNP referring to lanthanide NaYF4:Gd,Tb@NaYF4 scintillator nanoparticles and RB to rose bengal) (Figure 6a) synthesized through an in situ growth of a porphyrin Zr-MOF (−26 mv) on a lanthanide SNPs core-shell (+30 mv) (Figure 6b). The nanoprobe was further modified by the incorporation of RB with a high loading efficiency of ≈ 80% [138]. The process of X-ray excited luminescence (XEL) (Figure 6c), which results from the photoelectric effect and Compton scattering following a soft X-ray photon incidence, generates free electrons and holes in the inner core of heavy atoms (Tb^3+^). The free electrons and holes are thermalized into the valence and conduction bands, emitting XEL from the excited triplet state through radiative transition [138,179]. Core-shell SNPs XEL spectra showed four XEL peaks Tb^3+^ centered at 489, 546, 584, and 620 nm. Due to less surface quenching, the development of the NaYF_4_ shell intensified the XEL level about 1.4-fold. Further to that, the overlap of the Zr-MOF with SNPs XEL displayed a drop in XEL intensity spectra of SNPs@Zr-MOF within the visible range (450–600 nm), attaining a 76% FRET efficiency. The addition of RB (absorption bands between 450–650 nm) further reduced the intensity of SNPs@Zr-MOF@RB XEL spectra within the 450–600 nm region, evidencing the increase of FRET efficiency. The energy transfer from SNPs to Zr-MOF in the presence of soft X-ray irradiation and the addition of RB increased ROS production (61% reduction of 1,3- diphenylisobenzofuran (DPBF) solution absorbance intensity at 417 nm). Moreover, SNPs@Zr-MOF could even produce ROS in deep tissues (25% reduction at a 3 cm tissue depth), improving PDT efficiency. The efficient soft X-ray-induced ROS production by SNPs@Zr-MOF@RB nanoparticles (1 mg/mL) led to significant cytotoxicity to 4T1 cells in vitro, promoting an efficient PDT-ablation of cancer cells, thereby inducing a stronger ICD and subsequently an adaptative antitumor immune response. 4T1 tumor-bearing mice treated with SNPs@Zr-MOF@RB (3 mg/mL) and exposed to soft X-ray for 5 min (Figure 6d) displayed a higher tumor inhibition rate, thereby promoting severe cell apoptosis (Figure 6e). SNPs@Zr-MOF@RB stronger PDT-induced ICD, due to increased ROS generation, is supported by higher CRT expression (>40%) at a cell’s surface, enabling an efficient deep tissue antitumor treatment and tumor cell killing. As a result, increased ROS production also led to higher IFN-*γ*, IL-6, and TNF-*α* cytokine expression and consequently increased expression of CD8^+^ T cells and CD4^+^/Treg ratio as well as concurrent reversion of the immunosuppressive TME, thereby effectively inhibiting tumor development by turning a “cold” tumor into a “hot” one [138].

In a recent study, Wang et al. developed an Er^3+^-doped NaLnF_4_@MOF core@shell heterostructure to overcome weak PDT tissue penetration via UCL, transforming NIR light into UV/visible light. The NaLnF_4_ core was modified with 3, 4-dihydroxyphenylpropionic (DHCA) (zeta potential changed from +23.8 mV to −9.4 mV) to enable the coordination of residual carboxyl groups with Zr_6_ clusters, promoting the growth of a porphyrinic Zr-MOF shell. The upconversion shell was designed to reduce the distance between the NaLnF_4_ core and the MOF layer, which, when combined with a spectrum overlap of UCL Er^3+^ centered peaks (525, 542, and 655 nm) with Zr-MOF Q band absorptions (520, 554, 590, and 646 nm), enabled efficient resonance energy transfer (RET) (energy transfer efficiency = 56%). In comparison to NIR-irradiated MOFs, the RET from NaLnF_4_ to the MOF in NaLnF_4_@MOF (0.021 mg/mL) allowed for a considerable increase in ^1^O_2_ generation and, as a result, a more effective PDT cytotoxicity against CT26 cells under NIR laser irradiation (980 nm) for 10 min. PDT-mediated NaLnF_4_@MOF treatment of CT26 tumor-bearing mice exposed to a laser for 10 min significantly inhibited tumor growth and reduced tumor weight. H and E examination of tumor samples revealed necrosis and nucleus dissociation induced by PDT. PDT-induced apoptosis and necrosis could result in ICD that would synergize well with ICB. In a bilateral CT26 model, the combination of NaLnF_4_@MOF (8.4 mg/mL) PDT with α-PD-L1 (50 μg/mice) injection raised the amount of tumor-infiltrating CD45^+^ T cells, CD4^+^ T cells, CD8^+^ T cells, and NK cells in primary and distant tumors. The synergistic combination of PDT and immunotherapy resulted in the eradication of primary tumors (100% tumor inhibition), while distant lesions were also effectively suppressed (95% inhibition rate) [139].

In a distinct approach to MOF application in synergistic photodynamic therapy, Liu et al. developed a tumor-specific immune nanoplatform using a porphyrin-based Zr-MOF (PCN-224) coated with fused DCs and 4T1 cell cytomembranes, thereby producing an PCN@FM composite. The fused cell cytomenebrane (FM) coating changed the zeta potential from positive to negative, resulting in a negative charge on the nanoparticle surface. When compared to an uncoated PCN, the core-shell nanostructure of PCN@FM was shown to provide better stability in water and 10% serum medium, allowing the time in blood circulation to be extended and thus enabling more opportunities for accumulation at the tumor site. The 4T1 cell membrane proteins in the fused cell cytomenebrane (FM) coating allowed the nanoparticle to specifically target tumor cells of the same type, as well as faster endocytosis into the cell due to the strong adhesion among homotypic tumor cells Through a MTT assay, 4T1 cells incubated with 100 μg/mL (equivalent concentration of PCN) PCN@FM and PCN@CM (PCN coated only with 4T1 cell membrane) and subsequently irradiated with LED light (660 nm at 0.03 W/cm^2^ for 6 min), resulted in higher necrosis/apoptosis rates (5.69% and 6.20%, respectively) due to a stronger affinity and accumulation at 4T1 cells. On the other hand, PCN@FM may elicit a greater DC maturation and immune response due to the whole tumor antigens present in the FM inherited from the fused DC membrane. In vitro induction of the immunostimulatory activity of bone marrow dendritic cells (BMDCs) by PCN@FM resulted in greater intensity fluorescence of DC maturation markers CD80 (18.9%) and CD86 (61.0%). Using a bilateral subcutaneous 4T1-tumor-bearing mouse model, five different composites and PBS as a control were injected with an equal PCN concentration (100 μL, 6.4 mg/mL per mouse). Because of the increased immune response caused by the combination of PDT and immunotherapy, mice treated with PCN@FM under irradiation (660 nm, 0.4 W/cm^2^, 5min) could reduce and nearly eliminate primary and distant tumors. PCN@FM could even substantially restrict the growth of distant tumors without being subjected to irradiation due to the array of tumor antigens in FM and induced systemic immune response. Therefore, tumors collected 36 days after inoculation presented a higher accumulation of CD3^+^CD8^+^ T cells at distant tumors and higher expression levels of caspase-3. The administration of PCN@FM led to a survival rate of 40% of the six mice 70 days after tumor inoculation. The absence of abnormalities in major organs in H and E staining as well as normal blood physiological and biochemical indexes suggested a good biocompatibility of the nanocomposite. Therefore, Liu et al.’s nanodesign can be applied to various tumors for tumor specific synergetic photodynamic immunotherapy in cancer [126].

In another study, Chen et al. reported the synthesis of Apt/PDGs-s@pMOF (where Apt refers to periostin-targeting DNA aptamer and PDG refers to GEM-loaded DGLs shells) nanoparticles to enhance intratumoral CTL infiltration and reverse the immunosuppressive tumor microenvironment. Through a layer-by-layer method, the positive charged PDG adsorbed (zeta potential = +24.9 mV) to the negative surface of the porphyritic MOF via electrostatic attraction, further reinforced by the addition of a ROS-sensitive crosslinking. The nanoparticles were coated by Apt (zeta potential = −27.5 mV) for active targeting of tumor cells [137]. Gemcitabine (GEM) is a chemotherapeutic drug that inhibits MDSCs through the selective blockage of the JAK/STAT3 pathway, responsible for their formation, inhibiting the immunosuppressive effects in the tumor microenvironment [137,180,181]. Due to hydrolysis by an intracellular enzyme, Apt/PDGs-s@pMOF presented a high release ratio of GEM in 4T1 in just 6 h. In vitro treatment of 4T1 cells with LED light irradiation (660 nm, 0.03 W/cm^2^, 5 min) Apt/PDGs-s@pMOF (24 μg/mL pMOF concentration) resulted in an efficient PDT with an IC_50_ of 0.310 μg/mL. Moreover, Apt/PDGs-s@pMOF PDT increased CRT exposure (above 30%) as well as HMGB1 release and ATP extracellular secretion, all of which are indicators of a robust ICD induction and contribute to a higher DC maturation rate (nearly 20.5%) and immune response. The nanoparticle delivery depends on PDG’s “proton sponge” effect, which bursts lysosomes and releases them into the tumor cells. The cleavage of the crosslinking, activated by PDT-ROS production, is critical for PDG escape from the MOF surface and deeper penetration into the tumor lesion, resulting in the suppression of MDSC formation via the STAT3 pathway. As a result, when Apt/PDGs-s@pMOF was administered to bone marrow cells (BMCs) stimulated with IL-6 and granulocyte-macrophage colony-stimulating factor (GM-CSF), the percentage of CD11b^+^ Gr-1^+^, MDSCs decreased from 23% to 4.19%, indicating that the nanoplatform could enhance GEM inhibition of STAT3 phosphorylation and eliminate MDSCs. In addition, 4T1 tumor-bearing mouse models treated with Apt/PDGs-s@pMOF (10 mg/kg of pMOF) and subjected to a 660 nm laser (0.3 W/cm^2^) for 5 min showed strong antitumoral effects in vivo. Furthermore, crosslinking proved critical for both PDG delivery and for the antitumor activity of the nanoplatform, since a lack of crosslinking resulted in no tumor inhibition. Apt/PDGs-s@pMOF and light treatment resulted in a 40% decrease in MDSCs; this was consistent with in vitro studies. As a result, the combination of PDT-induced ICD and GEM immunosuppression inhibition improved DC maturation (from 5.48% to 11.6% in tumor-draining lymph nodes) and T-cell infiltration at the tumor site, as well as mobilizing a systemic body immunity (10.8% CD8^+^ T cells and 28.9% CD4^+^ T cells in the spleen), effectively inhibiting local tumor growth. Moreover, during the evaluation of the therapeutic effect on distant tumors in a bilateral tumor model, overexpression of CD3^+^ CD8^+^ and CD3^+^ CD4^+^ T cells in the blood, as well as the downregulation of MDSCs, resulted in substantial abscopal effects. Biochemical indexes and the absence of organ damage in H&E staining revealed that the nanoparticle therapy had negligible toxicity [137].

In general, the porous structure, wide surface area, and photo-responsive organic ligands of MOFs allow for the loading of therapeutic drugs and the direct production of ROS for an efficient PDT and synergetic cancer therapy. Most studies discussed in this review rely on the properties of prophirinic organic ligands irradiated with 630–670 nm lasers to generate and boost ROS generation without requiring any extra modifications. It is worth mentioning that an alternative chlorine-based MOF has been shown to boost PDT efficiency even further. Moreover, deep tissue PDT could be achieved by employing lanthanide-MOF core-shell structures exposed to either soft X-rays or light at 980 nm. PDT efficiency has shown to be improved by surface modifications aimed at specific tumor cell targeting, such as tumor cell membrane coatings, to promote homotypic targeting, resulting in higher accumulation at the lesion site and higher rates of endocytosis. As a whole, MOF-mediated combinatorial treatment elicited apoptosis in more than 70% of cells incubated with concentrations below 50 μg/mL in vitro, as well as more than 90% inhibition of primary tumor growth and regression in vivo. Most authors combined PDT with immunotherapy by either adsorbing the immunoadjuvant CpG to the surface of MOFs or employing ICB treatment, primarily by injecting α-PD-L1 at doses less than 75 mg per mouse. Studies that used a CpG modification demonstrated that PDT-induced ICD effects were amplified, promoting a considerable increase in DC maturation (over 60% in most cases). Therapeutic strategies that included ICB therapy yielded the best therapeutic outcomes by enhancing antitumor immune cell mobilization and infiltration at primary and distant tumors. As a result, in certain situations, combination therapy achieved more than 90% primary and distant tumor suppression, and even eradication in some cases. Accordingly, anticancer systemic mobilization may create long-term antitumor memory against tumor recurrence, extending survival duration and attaining 100% survival rates past 30 days after treatment in some studies. Furthermore, all studies corroborated the remarkable biocompatibility of MOFs, with nearly all treatments exhibiting negligible toxicity in mice.

Overall, the findings indicate that MOFs are a suitable nanoplatform for synergetic PDT-immunotherapy. The development of MOF-based approaches aimed at boosting ROS generation and increasing rates of ICD have shown to be an ideal tool for combination with diverse immunotherapeutic strategies to amplify an immune response. Furthermore, these nanomaterials addressed fundamental limitations of PDT and immunotherapy in cancer treatment. As a result, the tunability of MOFs enables the synthesis of various nanocomposites that can lead to superior therapeutic outcomes.

### 3.2. Synergistic Strategies of PTT and Immunotherapy

PTT involves the non-invasive ablation of tumors by generating heat in the tissue through photothermal conversion [118,182]. PTT, like PDT, has sparked interest because of its capacity to induce ICD. The development of a temperature gradient in the TME elicits several biological reactions within the cell that may induce ICD by enhancing antigen exposure and DAMP release. However, such an impact is frequently insufficient to stimulate an effective immune response to prevent recurrence and metastatic tumors. As a result, combining it with immunotherapeutic approaches might stimulate an immune response for a specific antitumor effect [117]. Additionally, PTT has other limitations: (i) NIR light has a low penetration depth; (ii) thermal agents are non-specific; and (iii) PTA photobleaching after a short period reduces PTT efficiency [115,118]. MOFs can overcome some of these constraints by increasing the photothermal efficiency and stability of PTAs while serving as nanocarriers for immunotherapeutic agents [81]. Accordingly, this section will discuss several strategies developed for synergetic cancer treatments of PTT and immunotherapy using MOFs, including PBNPs.

Zheng et al. developed a dual immunotherapeutic nanoplatform based on the MOF ZIF-8 for synergistic therapy by enhancing PTT and concurrently stimulating multiple cells in the antitumor immune process for synergistic immune amplification. The photothermal agent indocyanine green (IR820) was loaded into the ZIF-8 with a high loading percentage (34.4%). The nanocomposite surface was subsequently changed with HA to generate the composite HA/IR820@ZIF-8. The carboxyl and amide C=O groups of the HA structure were consumed by the coordination of HA with Zr ion in the ZIF-8 structure, slightly decreasing the zeta potential from −30.90 ± 5.88 mV to −37.40 ± 6.22 mV [120]. The addition of HA, a well-known ligand for the cell surface receptor CD44, which is highly expressed in tumors [183], enables specific target delivery and enhanced endocytosis of the composite into tumor cells. In vitro cellular uptake studies of murine melanoma cells (B16F10) treated with varied concentrations (0–100 μg/mL) of HA/IR820@ZIF-8 and IR820@ZIF-8 demonstrated that HA/IR820@ZIF-8 exhibits 2.86-fold uptake efficiency and greater accumulation at the tumor cells when compared to IR820@ZIF-8 group. Moreover, HA/IR820@ZIF-8 could increase the temperature above 30 °C at maximum concentration. The local accumulation of HA/IR820@ZIF-8 in the tumor cells enables a highly efficient PTT when irradiated with a laser at 808 nm (1 W/cm^2^) for 5 min, reaching the highest percentage of apoptosis (77.7%) at a concentration of 20 μg/mL. Simultaneously, due to the high percentage of apoptotic cells, a robust ICD was triggered, confirmed by DAMPs overexpression. Treatment with irradiated HA/IR820@ZIF-8 resulted in increased calreticulin (CRT) cell surface exposure as well as considerable upregulation and release of HMGB1 from cells. Concurrently, MAN/(R837+1 MT)@ZIF-8 was synthesized by encapsulating the immune adjuvant, R837, and immunomodulator, 1-Methyl-D-tryptophan (1 MT), in ZIF-8 with low-loading percentages of 8.9% and 10.7%, respectively. Because of the abundance of phosphate and hydroxyl groups in the mannan (MAN) structure, the surface modification of (R837+1 MT)@ZIF-8 with MAN, for targeted transport of the nanocomposite into dendritic cells (DCs), drastically lowered its zeta potential (−8.85 ± 6.63 mV to −36.80 ± 6.52 mV). The immunomodulator and adjuvant were delivered by MAN through a pH-responsive delivery system. When the composite was incubated in a PBS solution at pH 5, ZIF-8 dissolved and released about 100% of R837 and 1 MT content. After 24 h of incubation, flow cytometry analysis of DC maturation using bone marrow-derived dendritic cells (BMDCs) revealed that cells incubated with MAN/(R837+1 MT)@ZIF-8 had 7.40-fold higher fluorescence than the (R837+1 MT)@ZIF-8 composite, indicating higher DC maturation via upregulation of the CD80 and CD86 adjuvants and increased cytokine secretion. Furthermore, the ability of the immunomodulator 1 MT to block kynurenine synthesis in the indoleamine 2,3-dioxygenase (IDO)-pathway was evaluated in a BMDC incubation, resulting in a more than 50% inhibition at the highest concentration of MAN/(R837+1 MT)@ZIF-8 (19.4 μg/mL), thereby potentially avoiding immunological evasion and increasing T cell proliferation. The combined treatment with HA/IR820@ZIF-8 and MAN/(R837+1 MT)@ZIF-8 showed a potential synergistic impact on DC maturation and antitumor immunity. In vivo studies were performed on B16F10 cells-bearing mice with tumor volumes between 50 and 100 mm^3^. Irradiation with a laser at 808 nm (1 W/cm^2^) for 5 min, 6 h after injection of both composites, improved the antitumor immune response. HA/IR820@ZIF-8 could increase the temperature above 60 °C for an effective thermal ablation. The combination of PTT-triggered ICD and adjuvants boosted DC maturation, which, in conjunction with immunomodulation, increased CD4^+^ and CD8^+^ T-cell proliferation and infiltration. This strategy inhibited tumor growth by 97.7% and even eliminated the tumor in 40% (4/10) of the mice used in this group study while minimizing toxicity, as demonstrated by the absence of substantial morphological abnormalities on the organs following a histological examination. In addition, the combination of HA/IR820@ZIF-8 and MAN/(R837+1 MT)@ZIF-8 elicited a large systemic immune response, resulting in a considerable abscopal impact in distant tumors and immunological memory for rechallenged tumors [120].

Yu et al. rationally designed a practical and flexible MOF nanoplatform capable of integrating several therapeutic functionalities for site-specific delivery at the tumor site. The photothermal agent indocyanine green (ICG) and the immunological adjuvant imiquimod (IMQ) were encapsulated in ZIF-8 with an efficiency of 9.2% and 6.6%, respectively. The reduction in zeta potential from 25.8 ± 5.3 to −20.4 ± 1.8 mV confirmed the coating of HA to the ZIF-8@ICG@IMQ surface, resulting in the composite HA/ZIF-8@ICG@IMQ. HA facilitated targeted delivery and accumulation at tumor cells. The authors reported that the nanoplatform improved ICG’s photothermal stability and therefore its photothermal performance. The process of controlled release of ICG and IMQ levels was evaluated at various pH levels, as well as in the presence or absence of light irradiation (808 nm, 1 W/cm^2^, 5 min). The results have shown an acidic pH enhanced drug release. Under pH 5.5, 6.5, and 7.4, HA/ZIF-8@ICG@IMQ released 60%, 50%, and 20% of the ICG or IMQ, respectively. Furthermore, light irradiation accelerated the release of ICG or IMQ at pH 5.5 for an acidic pH/near-infrared (NIR) sensitive drug delivery system that enabled the controlled delivery of drugs to the tumor site, improving therapeutic efficacy while minimizing adverse effects. HA/ZIF-8@ICG@IMQ (150 μg/mL) could increase the temperature to nearly 55 °C. Under an 808 nm (1 W/cm^2^) laser irradiation for 5 min, different concentrations (0–80 μg/mL) of HA/ZIF-8@ICG@IMQ mediated satisfactory PTT, inducing antitumor effects in vitro, decreasing cell viability (under 40% at the highest concentration), and eliciting a 44.2% apoptotic ratio. Because of the combination TAAs, resulting from PTT, and the immune adjuvant IMQ, HA/ZIF-8@ICG@IMQ promoted maximum DC maturation. In a bilateral CT26 tumor model, in vivo injection of HA/ZIF-8@ICG@IMQ and laser irradiation (808 nm, 1 W/cm^2^, 5 min) could considerably suppress or eliminate primary or local tumors, reducing tumor weight to 0.2 g when compared to the PBS control group (1.4 g). Distant tumor growth was significantly suppressed due to the synergistic impact of enhanced DC maturation and subsequent immune response activation, resulting in tumor weight reduction (0.3 g). After therapy, there was a considerable rise in immune cytokines TNF-α, IL-6, and IFN-γ in peripheral blood serum, as well as a robust infiltration of CD8^+^ T cells at primary and distant tumor sites, resulting in systemic therapeutic antitumor effects. In mice treated with HA/ZIF-8@ICG@IMQ and NIR, the presence of endogenous CD8^+^ CD44^+^ CD122^+^ central memory T cells (TCM) increased (9.68%), enhancing long-term immunological memory effects against recurrent and rechallenged tumors. All five mice treated with irradiated HA/ZIF-8@ICG@IMQ treatment survived for more than 60 days. H&E staining and immunohistochemical assessment, supported by the unchanged weight of the mice, indicated no major abnormalities, confirming the lack of toxicity. This study emphasizes the importance of rationally designing nanoplatforms as potential nanotherapeutics for cold tumor therapy [122].

Ni et al. presented a novel strategy combining PTT and chemotherapy to trigger a strong antitumor immune response through an enhanced induction of ICD. During MIL-100/MTO/HA (where MTO refers to mitoxantrone) nanoparticles (NPs) or MMH NPs synthesis, BTC and FeCl_3_·6H_2_O were melted in *N*,*N*-dimethylformamide (DMF) to produce MIL-100. Subsequently, MIL-100 encapsulated mitoxantrone (MTO) with 88.7% loading efficiency at a 4/1.5 weight ratio. Nanoparticles were further modified with HA at a 3/2 weight ratio (<−20 zeta potential) for specific targeting of tumor cells. When irradiated with a 671 nm (1 W/cm^2^) laser for 5 min, MMH NPs demonstrated good photothermal stability and photothermal conversion efficiency (η = 16.2%), achieving high temperatures even at low concentrations (55 °C at 100 μg/mL and 43.5 °C at 25 μg/mL). Furthermore, MTO release was replicated in vitro at pH 7.4, 6.5, and 5.5. After 72 h, the proportion of MTO released increased significantly (>60%) under pH 5.5 conditions, indicating an acidic pH-triggered delivery system of MMH NPs. Further in vitro assays revealed a high antitumor efficacy as well as higher CRT expression (19.5 ± 3.5%) and HMGB1 release (91.2 ± 11.6%) from CT26 cells incubated with MMH NPs (2.5 μg/mL MTO concentration) and exposed to a laser at 671 nm (1.0 W/cm^2^) for 5 min, confirming the synergetic effect of PTT and chemotherapy for a stronger ICD induction. Such results were further demonstrated by immunohistochemistry experiments in vivo with CT26 cell-bearing mice. Treatment with MMH NPs (5 mg/kg of MTO concentration, irradiated with a laser at 671 nm (1.0 W/cm^2^) for 5 min yielded a 45.6% ± 3.2% of CRT exposure and 14.6% ± 3.9% HMGB1 release. The combined treatment of MMH NPs with OX40 (20.0 μg per mouse), an anti-OX40 antibody that decreases the immunosuppressive TME, demonstrated an excellent antitumor effect and tumor inhibition due to higher DC activation (5.0% ± 1.7%) and reduced immunosuppressive response (reduction to 2.0% ± 0.9% MDSCs and 0.4% ± 0.3% M2 macrophages), which resulted in greater CD4^+^ (4.5% ± 0.5%) and CD8^+^ (2.5% ± 0.5%) T cells infiltration in the tumor. This synergy-induced immune response likewise resulted in robust abscopal effects and metastatic reduction in a bilateral CT26 tumor model. MNN NPs are proposed as a MOF nanoplatform with multifunctional properties for synergetic therapies, with considerable potential for use in cancer therapy [133].

**Figure 7:**
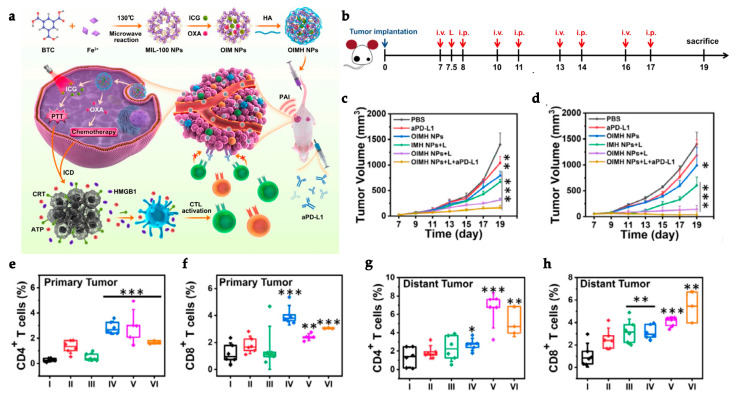
MOF-based synergetic PTT-immunotherapy strategy. (**a**) OIMH NPs were synthesized by loading ICG and OXA into MIL-100 NPs and subsequently coated with HA. OIMH NPs combine PTT with chemotherapy to ensure a robust ICD and subsequent antitumor immune response that can be synergized with ICB therapy. (**b**) CT26 cells were subcutaneously injected in mice and grown to a volume of 150 mm^3^. After day 7, tumor-bearing mice were divided into 5 treatment groups (*n* = 6): PBS (I), aPD-L1 (II), OIMH NPs (III), IMH NPs+laser (IV), OIMH NPs+laser (V) and OIMH NPs+laser+aPD-L1 (VI). Laser irradiation (808 nm, 0.8 W/cm^2^, 10 min) has been performed 12h post-injection. (**c**,**d**) Tumor volume variation of primary (**c**) and distant (**d**) tumors. (**e**–**h**) Percentage of tumor-infiltrating T Cells: e) CD4^+^ T cells in primary tumors; f) CD8^+^ T cells in primary tumors; (**g**) CD4^+^ T cells in distant tumors; (**h**) CD8^+^ T cells in distant tumors. Data are shown as mean ± SD (*n* = 6). *** *p* < 0.001, ** *p* < 0.01, * *p* < 0.05 [134]. Copyright © 2022, with permission from Elsevier.

Liu et al. developed a multifunctional MOF-based nanoparticle that might be used in a cancer therapy that combines chemotherapy, PTT, and immunotherapy. ICG and OXA were loaded into MIL-100 NPs using a one-step encapsulation process yielding loading efficiencies of 92% and 10%, respectively (Figure 7a). To offer long-term stability in physiological settings, the resulting OIM (where O refers to OXA, I to ICG, and M to the MIL-100) nanoparticles were further coated with HA (zeta potential = −30 mv) at an appropriate weight HA/OIM ratio of 0.25:1, resulting in OIMH. The addition of an HA coating resulted in long-term resistance to saline ions and proteins, allowing for blood circulation and tumor formation via the EPR effect. Under an 808 nm NIR laser (0.8 W/cm^2^) irradiation for 5 min, OIMH NPs at 40 μg/mL of ICG concentration raised the temperature to 64 °C for a photothermal conversion efficiency of 22.6% (compared to 14.7% for ICG); this is easily quenched under irradiation. After incubation with CT26 cells, OIMH reached a cellular content of 200 ng/10^6^ cells after 4 h. OIMH (20 μg/mL of OXA and 23 μg/mL of ICG concentration) presented high cytotoxicity of CT26 cells exposed to 808 nm NIR (0.8 W/cm^2^) irradiation for 5 min, displaying 90.4% apoptosis rate. Furthermore, increased CRT expression, HMGB1 leakage, and ATP release confirmed the amplification of ICD induction by the combination of PTT and chemotherapy. The synergetic therapy also elicited cytotoxic effects in vivo assays of CT26-tumor-bearing mice. The authors reported that administering OIMH NPs (2 mg/kg of OXA, 2.3 mg/kg of ICG) every 3 days, followed by 10 min laser irradiation (Figure 7b), resulted in rapid and effective tumor ablation and suppression. Furthermore, chemo-photothermal therapy-induced ICD increased TME immunogenicity and T cell activation, significantly boosting the number of CD4^+^ (30.9%) and CD8^+^ (31.3%) T cells at the tumor site. The use of OIMH NPs in combination with α-PD-L1 (2.5 mg/kg) in a CT26 bilateral mice tumor model led to increased infiltration of CD4^+^ (18.9% and 33.4%) (Figure 7e,g) and CD8^+^ (36.7% and 35.1%) T cells (Figure 7f,h) in primary and distant tumors, respectively, resulting in greater tumor growth inhibition (Figure 7c,d). Additionally, it was postulated that the rise in CD4^+^ (51.5%) and CD8^+^ (35.1%) in the spleen tissue would prevent metastasis and recurrence of the tumor [134].

Cano-Mejia et al. employed PBNPs in combination with anti-CTLA-4 checkpoint inhibition therapy for a synergistic photothermal-immunotherapeutic treatment of neuroblastoma. PBNP were synthesized in a co-precipitation method by mixing FeCl_3_6H_2_O with an aqueous solution of K_4_Fe(CN)_6_3H_2_O. While PBNPs showed that they can remain intact at an acidic (pH 5.5) and neutral (pH 7.0) pH, at slightly alkaline pH 7.4, which mimics blood and lymph pH, there was an apparent breakdown due to the potential assault of Fe^II^-CN-Fe^III^ by hydroxyl ions resulting in the formation of hydroxides and cyanoferrate. The degradation under alkaline pH has a direct impact on PTT efficiency, as a considerable drop in temperature was registered when compared to a pH 5.5 (60 °C, pH 7.4 versus 80 °C, pH 5.5) under 808 nm NIR laser at 1.875 W/cm^2^ for ten minutes. As a result, PBNPs demonstrated pH-dependent stability, with better stability at acidic and neutral pH, making it acceptable for administration in neuroblastoma PTT therapy. Upon treatment of Neuro2a tumor-bearing mice with 1 mg/mL of PBNPs and exposed to a laser at 808 nm (1.875 W/cm^2^) for 10 min, tumors shrank quickly; however, tumors recurred in a mean of 3 days after treatment. After 92 h, PBNPs-mediated PTT therapy was able to exert an immunostimulatory effect, boosting the infiltration of CD45^+^ (9.7% against 4.1% untreated) and CD3^+^ (6.2% vs. 2.2% untreated) cells to the tumor site. Nevertheless, this was regarded as insufficient to activate a strong immune response. When combined with CTLA-4 ICB treatment (150 μg of anti-CTLA-4/mice), the tumor was significantly suppressed and eventually eliminated. Moreover, this synergetic therapy promoted the survival of 55.5% of mice treated (*n* ≥ 5) when compared to 0% of PBNP-based PTT treatment. Anti-CTLA-4 administration enhanced immunological activation and mobilization of T cells (CD4^+^ and CD8^+^) of PTT, which is critical for better therapeutic responses. Rechallenging trials in mice treated with PTT and anti-CTLA-4 after 90 days of tumor-free survival revealed long-term immune protection against tumor recurrence, swiftly eradicating the rechallenging tumor [140].

**Figure 8:**
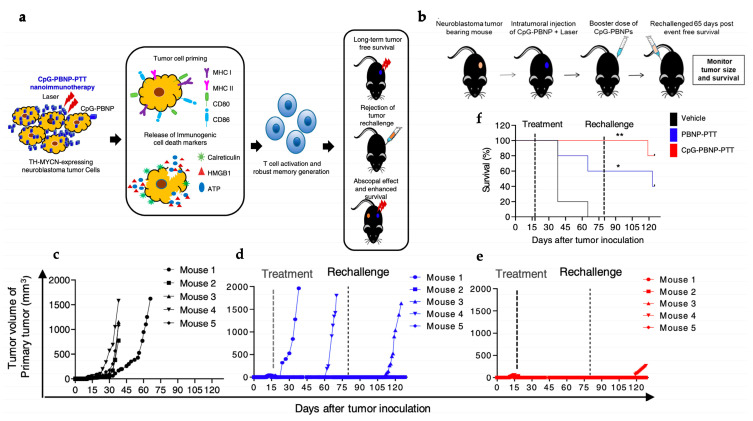
PBNPs synergetic PTT-immunotherapy strategy. (**a**) CpG-PBNPs photo-immunotherapy of MYCN overexpressing neuroblastoma cells enhances the antigen presentation by tumor cells as well as ICD triggering, promoting T cell activation and long term antitumoral memory effects. Treatment may promote long term tumor free survival, suppresses tumor rechallenging and elicits abscopal effects. (**b**) 9464D model cells were subcutaneously injected in mice and grown to a diameter of at least 5 mm. Tumor-bearing mice were divided into 3 treatment groups (*n* = 5): Vehicle (PBS), PBNP-PTT treatment and CpG-PBNPs-PTT treatment. Laser irradiation (808 nm, 1.5 W/cm^2^, 10 min) to a temperature of 120 °C. CpG-PBNP boosts without irradiation were injected on days 2 and 5. After 65 day of tumor free survival, the remaining mice were rechallenged to evaluate long term antitumor memory. (**c**–**e**) Tumor growth curves of tumor bearing mice treated with PBS (**c**), PBNP-PTT (**d**) and CpG-PBNP-PTT (**e**). (**f**) Survival plot of tumor bearing mice after treatment at day 18 with PBS, PBNP-PTT and CpG-PBNP-PTT and rechallenging at day 80. Data are means ± SD. * *p* < 0.05, ** *p* < 0.01. Adapted with permission from [141] © 2021 Wiley-VCH GmbH.

Shukla et al. employed a novel approach to combine PTT with immunotherapy for the treatment of neuroblastoma (Figure 8a). PBNPs were first coated with a positively charged polymer poly(ethylenimine) (PEI) (zeta potential increased) using a layer-by-layer coating process that facilitated the negatively charged CpG (zeta potential decreased) adsorption to the surface, resulting in CpG-PBNPs. Exposure to an 808 nm laser with a power range of 0.2–1.5 W/cm^2^ for 10 min revealed a laser power-dependent temperature increase reaching a high of 77 °C at a concentration of 0.15 mg/mL of CpG-PBNPs [141]. MYCN is an oncogene that belongs to a small family of genes that regulate the expression and transcription of several genes involved in cell proliferation, differentiation, metabolism, senescence, and apoptosis. In embryos, the expression of this gene is normally restricted by cells following the development of a nervous system. Tumors seem to mirror this pattern of expression, as MYCN is overexpressed in several neural tumors including neuroblastomas. In this study, the authors selected the 9464D neuroblastoma cell line due to a characteristic overexpression of the MYCN gene [184]. In vitro treatment with 0.15 mg/mL CpG-PBNPs-PTT irradiated with a 808 nm laser at a power of 1.5 W demonstrated the therapy’s ability to induce ICD, as cells displayed increased CRT expression at the cell surface and diminished intracellular levels of HMGB1 and ATP. When used in vivo in 9464D tumor-bearing mice, PTT raised the temperature to a maximum of 120 °C while exerting impressive ablating effects on the tumor at a dosage of 1 mg/mL and a potency of 1.5 W for 10 min. After CpG-PBNP-PTT therapy, 100% of the mice (5/5) exhibited total tumor regression (Figure 8c–e) and 100% survival after 80 days (Figure 8f). Furthermore, combined treatment provided long-term antitumor memory. At day 125 (45 days after tumor rechallenging), mice spleens showed a significant increase in CD3^+^ CD44^+^ memory cells, as well as a higher percentage of central memory CD4^+^ and both effector memory CD4^+^ and CD8^+^ T cells, indicating a robust immunologic memory induced by PTT, leading to a survival rate of 80% (4/5) after 125 days. The same authors reported that CpG-PBNP-PTT may also generate systemic antitumor immunity in bilateral tumor mice, slowing distant tumor development considerably [141].

In a different study, Cano-Mejia et al. used the CpG-PBNP nanoparticles employed by Shukla et al., and devised a combination with ICB therapy to enhance the presence of immunomodulatory agents on the treated tumor cells and potentiate abscopal effects. The PTT effect, as well as induced ICD, potentiated by the administration of CpG-PBNP-PTT and anti-CTLA-4 antibody (aCTLA-4) (at a concentration of 0.15 mg/mL of PBNPs, combined with irradiation by an 808 nm light at 0.75 W for 10 min and 20 μg/mL of aCTLA-4) in vitro in Neuro2a cells, stimulated the expression of antigens and molecular markers on the tumor cells; this, therefore, showed the potential to trigger immunostimulatory and immunosuppressive responses. The expression of CD86 (≈8%) increased 2-fold in PTT treatments in comparison to the control groups (untreated, laser, PBNP, and CpG) (≈3–4%) while CD80 (≈4–5%) remained constant. Similarly, the levels of MHC-II increased to nearly 11% (vs ≈ 6% in control), while MHC-I levels remained the same in all groups (≈3%). In contrast, PTT treatment was unable to increase the expression of PD-L1 (≈1%). On the other hand, B7H3, an immune checkpoint with immunosuppressive properties, was overexpressed (by around 10%), making it perfect for the application of ICB. Accordingly, in a bilateral synchronous tumor model of Neuro2a tumor-bearing mice, treatment with CpG-PBNP-PTT (1 mg/mL CpG-PBNPs irradiated by an 808 nm light (0.75 W) for 10 min and aCTLA-4 (150 μg of aCTLA-4), resulted in a complete regression of primary and distant tumors, promoting a higher survival rate of 55.5% of the mice after 60 days when compared to other treatments (0% for untreated, 0% in PBNP-PTT, 0% in CpG-PBNP-PTT, and 22.2% in PBNP-PTT + aCTLA-4). Furthermore, the combination of PTT, PTT-induced ICD, DC activation by CpG, and CTLA-4 immunosuppression reversal promoted long-term antitumor memory, as mice were capable of quickly eliminating rechallenged tumors induced 65 days after treatment [142].

Zhou et al. devised a strategy for the treatment of hepatocellular carcinoma (HCC) that combined PTT and sorafenib (SF) to promote the eradication of primary tumors while preventing metastasis and recurrence. Unlike typical PBNPs synthesis, which uses polyvinylpyrrolidone (PVP) as a stabilizer, gelatin was employed as a co-stabilizer, improving biocompatibility and facilitating modifications. Gelatin functional groups enable the thioester bond with the HCC-targeted SP94 peptide at the surface of the nanoparticles. PBNPs were further modified by loading SF to a loading content of 5% [143]. SF is a cancer therapeutic drug that inhibits the serine-threonine kinases Raf-1 and B-Raf, as well as the activity of vascular endothelial growth factor receptors (VEGFRs) and the platelet-derived growth factor receptor (PDGFR), thereby inhibiting cell proliferation, tumor angiogenesis, and promoting apoptosis [185]. SF was loaded with the assistance of 1-tetradecanol, a temperature-triggered (above 38 °C) gatekeeper of a drug delivery system. At a temperature of 42 °C, SP94-PB-SF-Cy5.5 swiftly released nearly 74% of the SF content. In contrast, only around 5% of the SF was released under different pH conditions (pH 4.5, 6.8, and 7.4). Hence, the acidic pH of the lysosomes and TME provides nearly no influence on SF release. The synthesis of SP94-PB-SF-Cy5.5 nanoparticles was completed by adsorption of Cyanine 5.5 (Cy5.5) to the surface via amide bonds, which conferred fluorescence imaging functionality. SP94-PB-SF-Cy5.5 (100 μg/mL) also demonstrated remarkable thermal stability, with a nearly constant temperature maintained during several cycles of irradiation with an 808 nm laser at 1.0 W/ cm^2^ for 5 min, reaching maximum temperatures of 47.47 ± 1.17 °C. The combination of SF and PTT improved the killing effect of the nanoparticle in human (HepG2) and mouse (Hepa1-6) liver cancer cell lines, reducing cell viability to less than 30% at a 200 μg/mL concentration. SP94-PB-SF-Cy5.5′s cytotoxic effects were further corroborated in mice with subcutaneous HepG2 tumors. SP94 improved tumor targeting and accumulation, resulting in a temperature increase during PTT (to 48 °C) after 15 min of SP94-PB-SF-Cy5.5 NPs (30 mg/kg of SF) under NIR (808 nm, 1.0 W/cm^2^, 15 min). Therefore, the combination with SF resulted in the complete eradication of the tumor (100% TGI) and a survival rate of 80% (4/5) after 60 days. The inherent ability of PB imparts catalase-like activity to nanoparticles, that then decompose excess H_2_O_2_ to create O_2_ in the TME, relieving hypoxia and thus easing TME immunosuppression. After treatment with SP94-PB-SF-Cy5.5 NPs and light, the percentage of M2 macrophages at the tumor site reduced considerably from 40.55 ± 1.77% in the PBS control to 10.85 ± 3.11%. Furthermore, PTT-induced ICD amplified CD45^+^CD3^+^CD8^+^ CTL recruitment to the tumor location (22.56 ± 2.18% vs. 7.48 ± 0.62% from the PBS group). To elicit a systemic antitumor immune response, the scientists combined SP94-PB-SF-Cy5.5 NPs and NIR treatment with anti-PD-L1 antibody (100 μg/mouse) in a bilateral Hepa1-6 HCC subcutaneous model. When compared to the SP94-PB-SF-Cy5.5 NPs and NIR single treatment model (16.12 ± 1.37% and 11.00 ± 1.16%), the results showed an increase in CD3^+^CD8^+^ CTL (25.37 ± 2.66%) and CD3^+^CD4^+^ T helper (Th) (18.20 ± 0.87%) at the distant tumor. After rechallenging assays, the spleen showed an increase in effector memory T cells (CD3^+^CD8^+^CD44^+^CD62L^−^) at day 40 (37.96 ± 4.82% vs. 15.72 ± 3.49%), indicating an antitumor immunological memory against recurrence. Therefore, the combined effects of hypoxia relief from PB, PTT, SF, and anti-PD-L1 extended the treatment outcome and effectively suppressed both distant metastatic tumors and recurrent cancers in addition to eradicating the primary tumors in an outstanding manner [143].

Balakrishnan et al. developed a novel treatment for melanoma combining PBNPs mediated PTT with agonistic anti-CD137 monoclonal antibody (mAB) therapy to reverse T cell immunosuppression and complement PTT-induced ICD effects in order to elicit a strong antitumor immune response. Similarly to previous papers, PBNPS were synthesized by a co-precipitation method through the mix of FeCl_3_6H_2_O with an aqueous solution of K_4_Fe(CN)_6_3H_2_O originating negatively charged nanoparticles (zeta potential = −30 mV). In vitro assays using PBNPs could reach a maximum temperature above 80 °C when exposed to an 808 nm laser at 2.0 W of potency for 10 min, presenting excellent thermal stability in water. However, that was not the case in complete cell culture media with 10% Fetal bovine serum (FBS), displaying a significant decrease in temperature after the first cycle of heating. SM1 murine melanoma cells were subjected to (0.15 mg/mL PBNPs) PBNPs-PTT with an 808 nm laser at 2.0 W/cm^2^ for 10 min, causing the apoptosis of more than 90% of the cells. Results also demonstrated an effective signal of ICD induction through upregulation of CRT (96.7%) while, due to the release of HMGB1, the intracellular percentage significantly diminished (37.7%). Moreover, the co-stimulation of immune cells by PBNPs-PTT could increase antigenicity and immunogenicity, increasing the expression of CD80 (mMFI (maximum mean fluorescent intensity) = 4.125), CD86 (mMFI = 768), MHC-1 (mMFI = 2557), and CD137 (mMFI = 155) antigens as well as a melanoma antigen that is recognized by T cells (MART-1/Melan-A mMFI= 725). Consequently, T cell activation (CD3+/CD69+) increased by 14.7%. The increase of CD137 in vitro provided a good opportunity to combine PBNPs-PTT with anti-CD137 antibodies. Therefore, in bilateral metachronous SM1 tumors, authors demonstrated that treatment with PBNPs-PTT-anti-CD137 (2.5 mg/kg PBNPs, 15 mg/kg anti-CD137, 808 laser at 2.0 W/cm^2^ for 10 min) eliminated 100% of primary tumors while significantly inhibiting the growth of distant tumors in 60% (3/5) of the mice treated. The combination of treatments also extended the mice survival rate to 60% at day 60 when compared to PBNP-PTT (20%) and aCD137 (0%) treatment groups. Similarly, in a bilateral synchronous tumor model, the treatment also eliminated 100% of primary tumors while significantly slowing distant tumor growth. Hence, synergy with anti-CD137 promoted systemic immune activation. The abscopal effect was driven by a higher DC maturation that elicited the increase of infiltrating CD8^+^ T cells (17%) in distant tumors. Consistent with these results, CD8 T cells were significantly increased in the spleen (64%). In contrast, CD4^+^ T cells percentage in the spleen diminished in comparison to untreated groups (CTRL) (47% vs. 59%); however, they appeared to be significantly more activated (9% vs. 5% of CD25 expression and 25.7% vs. 14% CD69 expression). Additionally, the nano-immunotherapeutic treatment could also increase the population of memory CD4^+^ (36%) and CD8^+^ (39%) T cells for the establishment of an immunological memory response against tumors. Remarkably, rechallenged tumors 66 days after treatment were rejected in 66% of the treated mice. Despite the impressive antitumor effects, the combination with aCD137 exerted acute hepatitis and significantly increased the inflammatory foci (142 vs. 8 in CTRL). However, such effects did not confer long-term liver health concerns as the mice could recover to normal-age liver health [144].

Most PTT studies exploited ZIF-8 and MIL-100 as nanocarriers for exogenous photothermal agents, such indocyanine green and mitoxantrone, that were irradiated with 808 nm and 671 nm lasers, respectively. PBNPs were reported as the only MOF with intrinsic photothermal properties, demonstrating a shortage of MOFs with such performance. In a few experiments using ICG encapsulation, the exogenous PTA raised the temperature over 55 °C, resulting in a photothermal conversion efficiency of 22.6% in one of the studies. Similarly, mitoxantrone achieved temperatures of 55 °C, although it had a lower photothermal conversion efficiency (16.2%). PBNPs, on the other hand, were shown to be able to reach a maximum temperature of 120 °C. HA modification was frequent in several composites and was proven to have a key role in PTT efficiency, promoting higher accumulation at the tumor sites. Overall, most investigations employing the carriers ZIF-8 and MIL-100 demonstrated that the different composites could trigger apoptosis in cells at concentrations ranging from 20 to 80 μg/mL in vitro, resulting in considerable tumor suppression and tumor volume shrinkage in in vivo models. Combination with ICB therapies has been proven, in a few trials, to further enhance a systemic antitumoral immune response, providing considerable abscopal effects and avoiding tumor recurrence. Notably, in contrast to most trials, one of the best outcomes was obtained without the use of ICB treatments. The combination of HA/IR820@ZIF-8 and MAN/(R837+1 MT)@ZIF-8 elicited a strong antitumor immune response, resulting in 97.7% tumor inhibition and full tumor eradication in 40% of the mice, as well as nearly complete suppression of distant tumor and tumor recurrence. To date, the most often reported modification of PBNPs in photo-immunotherapy has been CpG adsorption on the surface. When combined with ICB therapy, most PBNP-based therapies resulted in total eradication of primary tumors as well as suppression or regression of distant cancers. Furthermore, all therapies have shown to boost memory T cells, which provide antitumor memory against tumor recurrence. Remarkably, the vast majority of PBNP-based therapies promoted mice survival rates above 60% at 60 days after treatment.

The previous studies demonstrated how MOFs can be successfully employed in cancer synergetic PTT-immunotherapy. MOFs increased PTA stability, resulting in better photothermal conversion efficiencies and temperatures, as well as stronger tumor cell ablation and a significant ICD induction that synergized well with immuno-therapeutic methods. Nonetheless, the application of MOFs in synergetic PTT-immunotherapy is still rather unexplored.

### 3.3. Synergetic Dual-Photo-Immunotherapeutic Strategies

The lack of oxygen inside the TME substantially restricts PDT treatment in hypoxic tumors. PTT, on the other hand, requires complex modification to optimize photothermal efficiency [115,186]. In addition, higher laser power is necessary to improve ROS production and attain higher temperatures, possibly harming nearby healthy tissues [187]. As a result, synergistic therapy with PTT and PDT might compensate for the other’s limitations to achieve superior therapeutic effects [115]. PTT-induced temperature increase can promote blood flow and oxygen delivery to the tumor location, hence benefiting PTD. PDT, on the other hand, can enhance tumor sensitivity to temperature by interfering with the TME’s physiological properties [188]. This section examines “all-in-one” approaches that use MOFs as a nanoplatform for synergistic photo-immunotherapy.

In a recent effort to develop precise immunotherapy guided by multimodal imaging, Fan et al. described the design of a multimodal imaging-guided synergistic cancer photoimmunotherapy nanocomposite based on the MOF MIL-101. The adsorption of photoacoustic and fluorescent signal donors (indocyanine green, ICG) and immunological adjuvant (CpG ODN) in MIL-101, modified with an amino group (NH_2_), resulted in a reduction in the MOF’s zeta potential to synthesize ICG-CpG@MOF. CpG was loaded into ICG-MIL101-NH_2_ with a loading efficiency of 76% at a concentration of 65 μg. CpG was released to the tumor cells via a concentration-dependent glutathione (GSH) delivery mechanism. High GSH concentrations in the tumor microenvironment break down the MOF and release CpG. The absence of an adsorption balance in the high-pressure region of the nitrogen adsorption elution experiment indicated that the material had a large pore; this led the authors to speculate that CpG ODN could be adsorbed to the interior of the microporous structure and could only be released after MOF structure decomposition triggered by GSH concentration. The cytotoxic effects of ICG-CpG@MOF in 4T1 cells were investigated by incubating 4T1 cells with a 0–50 nM concentration of irradiated ICG-CpG@MOF (808 nm at 1.0 W/cm^2^ for 5 min). ICG’s photothermal and photodynamic effects in 50 nM ICG-CpG@MOF significantly reduced cell viability. Furthermore, treatment with 20 nM ICG-CpG@MOF followed by 5 min light irradiation at 808 nm (1.5 W/cm^2^) induced apoptosis in 70% of tumor cells. CpG administration treatment in isolated spleen cells increased CD80 and CD11c expression in adherent cells (49.07%). In contrast, 4T1 cells inhibited the expression of CD80 and CD11c (16.49%), although the expression increased following ICG-CpG@MOF irradiation (65.38%). Furthermore, 4T1 photothermal treatment with the MOF composite can induce T cells killing effect while also releasing immune cytokines, resulting in an immunological regulatory impact against tumors. ICG-CpG@MOF treatment involves a combination of immune adjuvant administration, PTT, and PDT. Results from in vivo experiments on mice bearing 4T1 tumor cells shown that the tumor vanished at day 21 after receiving subcutaneous injections of ICG-CpG@MOF (equal dosages of 4.5 g of CpG ODN) and laser irradiation (808 nm at 1.5 W/cm^2^) for 5 min. The five mice that received ICG-CpG@MOF and irradiation survived to the end of the study (+40 days), culminating in a 100% survival rate. A second injection of tumor cells after 21 days revealed that the treatment significantly reduced the risk of metastasis. The H and E-stained organs displayed no apparent damage in histocompatibility assays. Furthermore, ICG-CpG@MOF buildup in the liver and kidneys caused no evident harm, indicating low toxicity and side effects. As a result, this strategy is promising as a multi-synergetic cancer therapy [132].

**Figure 9:**
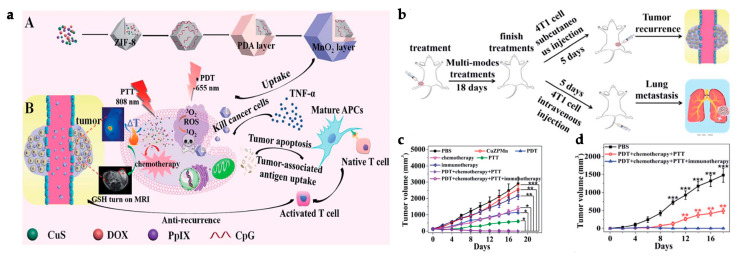
MOF-based synergetic “all-in-one” strategy. (**a**) CuZPMn@PpIX/DOX/CpG was synthesized by encapsulation of CuS nanoparticles, PpIX, and DOX into ZIF-8 CpG adsorption. PDA coating has been formed to facilitate a MnO_2_ layer growth. (**b**) 4T1 cells were injected into mice and grown to a volume of 100 mm^3^. Mice (*n* = 3) were treated with chemotherapy, PDT (655 nm), PTT (808 nm), and immunotherapy every 3 days within 18 days. 4T1 cells were injected 5 days after treatment in an anti-lung metastasis model as well as in a rechallenged tumor. (**c**) and (**d**) Tumor volume of primary (**c**) and recurrent (**d**) tumors [121].

Yang et al. reported the development of a flexible one-pot method combining multiple probes and agents into a single nanoplatform for controlled drug delivery in PTT, PDT, chemotherapy, and immunotherapy combination therapy (Figure 8a). CuZPMn@PpIX/DOX/CpG, an “all-in-one” system, was developed by encapsulating CuS nanoparticles (NPs) in ZIF-8 in a one-step loading strategy to promote a high photothermal conversion efficiency in PTT and NIR triggered drug release, followed by loading protoporphyrin IX (PpIX) for PDT and doxorubicin (DOX) for chemotherapy in a ZIF-8 MOF with a loading efficiency of 94 and 92%, respectively (Figure 9a). The nanoplatform was further modified for immunotherapy by adsorption of the negatively charged immune adjuvant CpG to the ZIF-8 positively charged surface with 98% loading efficiency. Such high loading efficiencies are dependent on ZIF-8′s encapsulation capability, as well as strong π–π stacking, hydrogen bonding, and electrostatic interactions between DNA (negatively charged) and ZIF-8 (positively charged). The nanocomposite was further coated with polydopamine (PDA) to improve PTT, protect CpG activity, and facilitate the generation of uniform MnO_2_ nanosheets on its surface for multimodal imaging and oxygen generation. A photothermal and pH-dependent mechanism controls the drug delivery system, causing nanocomposite breakdown and the release of CpG and DOX inside tumor cells. DOX was released as much as 2.8%, 7.6%, and 23.2% without NIR irradiation at pH 7.4, 6.5, and 5.0, respectively. In comparison, CuZPMn@DOX released 58.6%, 63.9%, and 71.2% of DOX after 4 h when exposed to NIR laser light at 808 nm (2 W/cm^2^) for 5 min. Therefore, the presence of acidic pH melted the ZIF-8 core, whereas the shell disintegrated following NIR stimuli due to CuS NPs and PDA local hyperthermia. For 4T1 cells incubated with 200 μg/mL of CuZPMn@PpIX/DOX/CpG and subjected to 808 and 655 nm lasers (2 W/cm^2^) for 10 min, the combination of PDT-PTT-chemotherapy-immunotherapy resulted in high tumor cytotoxicity, with a cell viability of less than 2% being found. Furthermore, 4T1 tumor-bearing mice, treated every 3 days with CuZPMn@PpIX/DOX/CpG and exposed to 808 and 655 nm lasers (2 W/cm^2^) for 10 min (Figure 8b), saw the total eradication of primary tumors (100% tumor inhibition) (Figure 8c) as well as consolidation of the therapeutic effect through the complete suppression of recurrence and metastasis tumors (Figure 8d); this resulted from the combined action of PTT, enhanced PDT, chemotherapy, and immunotherapy [121].

The previously discussed studies primarily relied on the usage of nanocarriers ZIF-8 and MIL-101 to load numerous therapeutic agents into a combination of PDT, PTT, and immunotherapy treatments. Both publications [121,132] employed MOFs that were modified with the immune adjuvant CpG for an immunotherapeutic effect, amplifying DC maturation; this was supported by an increase in CD80 and CD11c expression to approximately 65% in one study and by high cytokine output in the other. Fan et al. demonstrated that ICG had an additional photodynamic capacity enabling the combination of PDT and PTT therapeutic effects in a nanoplatform using an 808 nm laser. Accordingly, the impacts of PDT, PTT, and immunotherapy in vitro triggered apoptosis in 70% of the cells at low concentrations of 20 nM, as well as full eradication of primary tumor after 21 days of treatment, culminating in a survival rate of 100% at 40 days after treatment. Yang et al. employed CuS as a photothermal agent and PpIX as a photodynamic agent when exposed to 808 and 655 nm lasers, respectively. Notably, the combination of PTT, PDT, immunotherapy, and chemotherapy achieved the greatest therapeutic outcome, with cell viability reduced to 2% in vitro at a dose of 200 μg/mL. In vivo therapies, on the other hand, resulted in primary tumor elimination as well as total inhibition of tumor recurrence and metastasis.

Overall, MOFs have the potential to be a powerful multitherapeutic nanoplatform, allowing the combination of PDT, PTT, and immunotherapy in a single treatment. PDT and PTT synergy showed to have complimentary effects, allowing them to overcome inherent shortcomings of the therapies while also inducing a more robust ICD that synergized effectively with the effects of immunotherapeutic agents. Furthermore, these studies demonstrated MOF loading capabilities; this may serve as inspiration for new photo-immunotherapeutic approaches in cancer treatment.

## 4. Challenges and Opportunities

The study of MOF applications in the context of synergistic phototherapy and immunotherapy strategies for cancer treatment is still relatively new and unexplored. Owing to their unique properties and versatility, MOFs have sparked a surge in interest in PIT and synergistic photo-immunotherapy applications in recent years. The tunability of MOFs allows the development of nanoplatforms with intrinsic photo-responsive characteristics that can function as PSs and PTAs while inhibiting self-quenching and aggregation. Furthermore, the distinctive porous structure with several active sites, a large surface area, and a stimuli-responsive structure enable the simultaneous loading and delivery of different therapeutics, such as immunotherapeutic and chemotherapeutic agents, for potential synergistic therapies while minimizing potential toxicity. MOFs can play a pivotal role in PDT and PTT efficacy in cancer as well as in the efficiency of the delivery of therapeutic molecules into the TME. As a result, MOFs characteristics allow for the development of numerous synergistic approaches to overcome the drawbacks of both phototherapy and immunotherapy in cancer treatment (e.g., hypoxic TME, immunosuppressive TME, PSs, and PTAs stability and poor selectivity of immunotherapeutics). Several authors exploited MOFs properties to rationally design MOF-based nanoplatforms, seeking to improve both PDT and PTT efficiency (e.g., modifications enabling selective tumor cell targeting) while also amplifying immunotherapeutic effects (e.g., immune adjuvants loading). Most studies combining PDT and immunotherapy relied heavily on synergy with ICB treatment to get the highest therapeutic effects, with tumor inhibition rates above 90% and considerable protection against metastasis and tumor recurrence. On the other hand, PTT-immunotherapy proceeded to the near eradication or even complete elimination of primary tumors as well as successful suppression of distant and tumor recurrence, resulting in survival rates exceeding 60% at 60 days post-treatment. Nonetheless, the best outcomes could be attained using an “all-in-one” strategy through the loading of molecules with both photothermal and photodynamic properties combined with other immunological strategies, leading to the eradication of locally treated tumors as well as the elimination of metastatic and recurrent tumors. It is important to note that MOFs also provide remarkabley low toxicity during the therapy, as nearly all the studies reviewed reported a negligencial toxicity from the treatments. Table 2 summarizes the current knowledge reagarding the use of MOFs in synergistic cancer photo-immunotherapy.

Despite this, MOFs have several drawbacks, the major one being the challenge of developing these nanomaterials with suitable stability for biomedical applications. Moreover, the scarcity of alternative organic ligands with photo-responsive characteristics for use in PDT and PTT restricts the development of MOFs with intrinsic photo-responsive features. As a result, the alternatives for phototherapy MOFs remain somewhat restricted to porphyrin-based MOFs in PDT and the incorporation of exogenous PSs and PTAs into ZIF-8 and MIL MOFs for application in PDT or PTT. Additionally, because of absorption in the lower NIR region, the usage of porphyrin-based MOFs may be detrimental to PDT therapy efficacy of deep tissue tumors.

Combined treatment of cancer with phototherapy, immunotherapy, and even chemotherapy using MOFs nanoplatforms revealed high targeting ability, low drug dosage, and non-invasiveness, revealing the potential of MOFs in real-world cancer therapies as a good alternative or complement to traditional therapies. In addition to the benefits of MOFs in phototherapies, which have previously been widely covered, carrying and delivering immunotherapeutic and chemotherapeutic drugs has the potential to offer a more complete and efficient cancer therapy whilst reducing side effects. Immunotherapies such as checkpoint blockade have already provided significant advancements in cancer therapies. Anti-CTL4 and anti-PD1 antibody therapies have already led to complete therapeutic effects in several cancers, such as melanoma, prostate cancer, breast cancer, lung cancer, and ovarian cancer, among others. While such therapies provide good cancer regression and elimination, immunotherapies still lack specificity, promoting systemic inflammation and a wide range of autoimmune side effects (e.g., gastrointestinal toxicity, pruritis, fatigue, hypothyroidism, and hyperthyroidism) [189]. Furthermore, in chemotherapy, several chemotherapeutic agents, such as anthracycline agents, a family of anti-tumor antibiotics approved by the FDA, are also employed in cancer therapies. This family of drugs includes the aforementioned DOX, which is widely used in the treatment of breast, ovarian, stomach, lung, and liver cancers, as well as lymphomas and leukemia. However, despite being effective in cancer treatments, in certain instances chemotherapeutic drugs need high dosages for efficient tumor cell killing, resulting in severe toxicity and side effects and even restricting therapeutic application to the patients. In addition, certain cancers are drug resistant, which reduces drug efficacy [190]. Therefore, MOF-specific targeting and controlled release can enhance accumulation at cancer cells, reducing the drug dose required for efficient therapy, reducing toxicity, and enabling the combination with other treatments for more successful therapeutic outcomes. MOF biosensing and bioimaging properties may also be employed in cancer diagnosis (sensing cancer biomarkers, ions, and physicochemical parameters (pH, oxygen concentration, and temperature)) as well as allowing for real-time treatment monitoring via magnetic resonance imaging (MRI), optical imaging, and X-ray computer tomography [191]. As a result, MOF enable the development of theranostics systems, such as the one reported by Fan et al. [132], with the promise for more efficient and accurate diagnosis and cancer treatments.

**Figure 10:**
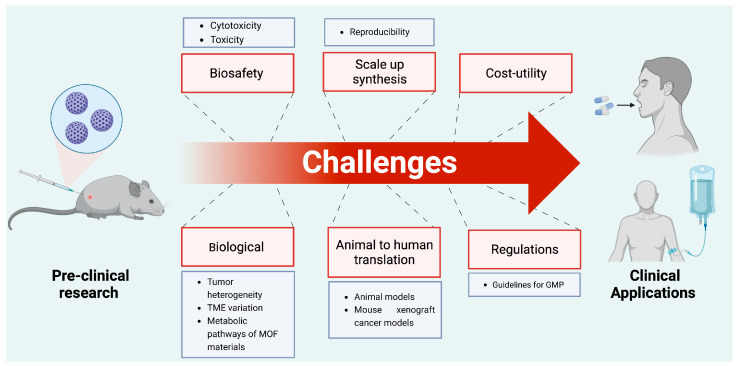
A summary illustration of the major clinical translation challenges of MOF-based treatments. Clinical application hurdles include biology, biosafety, animal-to-human translations, scaling up manufacturing, regulatory, and cost-utility issues. Created with BioRender.com.

Despite the growing attention in recent years and the evident capacity to successfully suppress tumors, there are still several challenges that must be solved before MOFs synergistic therapies can be employed in clinical settings: biological challenges, biosafety, scale up synthesis, regulations, and cost-utility [192,193]. Figure 10 summarizes the primary challenges for the clinical application of MOF-based therapies in cancer therapy. 

Biological challenges are major limitations for the clinical translation of MOF therapies. Most MOF studies focus on the uptake of the nanoplatforms, yet there is a lack of understanding of the mechanisms of MOF breakdown and clearance within tumor cells. Hence, the metabolic routes of the MOF materials should be elucidated in more detail [194]. Furthermore, tumor heterogeneity from patient-to-patient, as well as varying TME conditions (blood flow, pH variation, and oxygenation), can be a challenge for targeted drug delivery, decreasing the therapeutic efficiency [195]. Thus, the conditions of each tumor should be thoroughly studied to understand and apply the most suitable MOF composite. 

In vivo toxicity is yet another important limiting factor for the clinical application of nanomaterials. Despite promising results suggesting minimal cytotoxicity and toxicity of MOFs, there are still limited in vivo toxicity studies on different MOF materials [191,194]. Even so, preliminary studies identified that parameters such as size, shape, functionalization, and solvent systems may influence MOFs biodistribution and toxicity, as well as the use of toxic metals in the composition, such as lead, arsenic, cadmium, and chromium, which may promote severe health issues [196]. Nevertheless, further comprehensive and in-depth research of the long-term impacts of MOFs and their combined therapies may be necessary to better determine their biosafety.

Pre-clinical research seeks to clarify the mechanisms of action, drug delivery systems, and treatment safety, as well as the efficacy and stability of nanomedicines in cancer therapy, in order to prevent potentially expensive limitations that might hinder future investments and developments [197,198]. As a result, selecting suitable pre-clinical models is critical for understanding the effects of nanomedicine therapies on the immune system and lowering the risks for clinical applications. Although rodent models are the most commonly used to research immune responses to treatments, there are significant differences between them and human immune responses in terms of immune cell development, activation, complexity, proliferation, and function [198]. Moreover, the commonly used mouse xenograph models tend to overestimate nanomedicine’s effectiveness due to increased accumulation at the tumor site induced by an exaggerated EPR effect [197]. 

Finally, for clinical applications, MOFs must also comply with some requirements, such as reproducibility of the synthesis for scale up production, must meet the guidelines for good manufacturing practice (GMP), and find a balance between quality, complexity, cost, and effectiveness [193].

MOFs are versatile and unique materials with a vast number of possible compositions and topologies. However, versatility can raise challenges in understanding and controlling both structural and compositional complexity [199]. Despite amazing progress in recent years, there are still some fundamental issues, particularly in MOF functionalization, which restricts their development and efficacy for therapeutic applications. Many approaches for functionalization have been devised, although the majority of these methods are still flawed. The establishment of weak interactions between the MOFs and the incorporated molecules during adsorption and encapsulation approaches causes progressive leakage. On the other hand, immobilization through covalent binding methods may result in stronger interactions, although sophisticated procedures are required, and covalent links may prevent molecules from functioning as intended. While MOF functionalization by the incorporation of biomolecules, such as amino acids and peptides, as organic ligands promotes improved biocompatibility, biomolecules are more flexible than conventional organic ligands, rendering the creation of good quality MOFs more complex [200]. As a result, there is a need for novel or optimized functionalization methods that allow for the incorporation of a range of therapeutics to produce more suited and sophisticated MOFs for therapeutic applications. Moreover, the pharmacokinetics, degradation mechanisms, and toxicity of MOFs are still poorly understood, and further research is needed to develop and design novel MOF nanocomposites with higher stability, biocompatibility, and therapeutic performance [196,200]. Validation and standardization of testing procedures are also critical for the field's development [191].

Ultimately, research efforts in the field of MOF-based therapies should result in the development of more sophisticated and programmable MOF nanocomposites that support multiple functionalization for the combination of diagnosis and multiple therapies in a single MOF, aiming for the effective eradication of diverse primary and metastatic tumors as well as the prevention of recurrence, with minimal toxicity and side effects.

## 5. Conclusions

In this review we presented a comprehensive in-depth discussion of the MOFs up-to-date applications in synergistic cancer therapy. MOFs proved to provide good nanoplatforms for synergistic photo-immunotherapies of cancer, enhancing phototherapy efficacy and ICD induction as well as a good performance as nanocarriers of immunotherapeutic and chemotherapeutic agents, with controllable delivery systems. Despite promising results, most of the studies still fail in the complete eradication of tumors. Moreover, MOFs require more in-depth studies of their properties, and the several limitations in synthesis and functionalization need to be addressed. Nonetheless, MOFs hold great potential for cancer therapy. The rational design of different structures, employing different metal nodes and organic ligands, as well as the therapeutics loaded, may allow the development of synergetic techniques that surpass existing therapeutic limitations and provide more remarkable outcomes in the coming years.

## Figures and Tables

**Table 1:** MOF-based strategies for synergistic cancer photo-immunotherapy [120,121,122,123,124,125,126,127,128,129,130,131,132,133,134,135,136,137,138,139,140,141,142,143,144].

Original MOFs/Metal Node	MOFs Composites	Further Modifications	Particle Dimensions	Irradiation In Vivo	Tumor Cell Models	Photo/Chemo/Immunotherapy		Ref
						Mechanism	Efficacy	
ZIF-8/ Zn^2+^	HA/IR820@ZIF-8	IR820 adsorption to ZIF-8 surface + HA coating	120 nm	Laser PTT: 808 nm, 1 W/cm^2^, 5 min	B16F10	HA tumor targeting and ICD↑DAMPs MAN targeted DC delivery↑DC maturation and antitumoral response	↓97.7% tumor growth inhibition Systemic anti- metastatic response Immunologic memory	[120]
	MAN/(R837+1MT)@ZIF-8	Immune adjuvant R837 and immunomodulator 1-MT adsorption to ZIF-8 surface + MAN coating	221 nm					
ZIF-8/ Zn^2+^	CuZPMn@PpIX/DOX/CpG	CuS nanoparticles + PpIX + DOX encapsulation into ZIF-8 + CpG adsorption + PDA and MnO_2_ nanosheets coating	120–150 nm	Laser PTT: 808 nm, 2 W/cm^2^, 10 min Laser PDT: 655 nm, 0.2 W/cm^2^, 10 min	4T1	CuS and PDA coating↑PTT MnO_2_ O_2_ generation + PpIX↑PDT PTT, PDT, DOX chemotherapy + CpG immunotherapy synergistic effect	Primary tumors eradication No recurrence or metastasis	[121]
ZIF-8/ Zn^2+^	HA/ZIF- 8@ICG@IMQ	ICG and IMQ encapsulation + HA coating	134 nm	Laser PTT: 808 nm, 0.1 W/cm^2^, 5 min	CT26	ICG↑PTT TAAs release + IMQ ↑DC maturation and antitumoral response	Elimination of primary tumors Distant tumor growth inhibition Tumor recurrence prevention	[122]
PCN-224/Zr^4+^	M@O-A	OXA encapsulation + aptPD-L1 adsorption	139.1 nm	LED PDT: 640 nm; 0.1 W/cm^2^, 30 min	MC38	aptPD-L1 specific targeting of PD-L1-positive tumor cells PDT + chemotherapy ↑ICD and antitumor immune response Synergy with ICB	Tumor growth inhibition Longer survival rates Complete distant tumor inhibition	[123]
PCN-224/Zr^4+^	msiPCN	sicdk4 -protamine encapsulation + CT26 cell membrane coating	≈150 nm	He−Ne laser PDT: 660 nm, 0.1 W/cm^2^,2 min	CT26	Tumor cells homotypic targeting Cdk4 inhibition + PDT↑ICD, antigens release and PD-L1 expression Synergy with anti-PD-L1 anti-bodies	Tumor cell cycle arrest Tumor proliferation inhibition 100% survival rate after 30 days	[124]
PCN-224/Zr^4+^	MnO_2_@CPCN	CCCP encapsulation + MnO_2_ shell and PAH coating	117.6 nm	Laser PDT: 660 nm, 0.2 W/cm^2^, 10 min	4T1	MnO_2_ shell↓tumor hypoxia and↑ PDT efficiency MnO_2_ shell GSH scavenge releases CCCP↑pro death Mitophagy PDT + CCCP synergy ↑ICD , autophagy and antitumor immune response	100% survival rate Tumor growth inhibition Tumor tissue eradication in 20% of mice Prevention of tumor metastasis and recurrence	[125]
PCN-224/Zr^4+^	PCN@FM	FMs (DC cells + 4T1 cells) coating.	≈175 nm	Laser PDT: 660 nm, 0.4 W/cm^2^, 5min	4T1	FM coating tumor homotypic targeting PDT↑ICD and antigen production FM coating + PDT synergistic effect	Primary tumors rebound proliferation inhibition Distant tumors proliferation suppression 70 days survival in 40% of the mice	[126]
PCN-224/Zr^4+^	PCN-ACF-CpG@HA	ACF and CpG adsorption + HA coating	105.4 -117.5 nm	Laser PDT: 670 nm, 0.25 W/cm^2^, 10 min	H22	HA tumor cells specific targeting PDT↑ICD and antigen release PDT+ CpG↑DCs maturation ACF HIF-1α inhibition ↓immunosuppression	Tumor growth inhibition and cell destruction Metastasis inhibition	[127]
W-TBP/W^6+^	W-TBP/CpG	CpG adsorption	Diameter: 114.0 ± 6.7 nm Width: 100 nm Length: 200 nm	Light PDT: 650 nm, 0.1 W/cm^2^, 7.5 min	TUBO	PDT↑ICD and TAAs PDT+ CpG↑DCs maturation Synergy with ICB	96.6% tumor regression Abscopal effects when synergizing with ICB	[128]
Fe-TBP/Fe^3+^	Fe-TBP	________	100 nm in length	LED PDT: 650 nm, 0.1 W/cm^2^, 7.5 min	CT26	Fe-TBP Fenton-like reaction ↑O_2_ and ↑ PDT PDT↑ICD Synergy with ICB	>90% regression of primary and distant tumors	[129]
Pd-TBP/Pd^2+^	PTP@M	4T1 cell membrane coating	165 nm	Laser PDT: 630 nm, 0.3 W/cm^2^, 5 min	4T1	4T1 cell membrane coating tumor cell homotypic targeting π-extended Pd-TBP in PTP ↑ PDT and ↑ ICD Synergy with ICB ↑ antitumor immune response	Tumor inhibition Anti-metastasis effect	[130]
Cu-TBP/Cu^2+^	Cu-TBP	________	164.1 ± 48.5 nm	LED PDT: 650 nm, 0.1 W/cm^2^, 30 min	B16F10 and SKOV-3	pH dependent release of Cu^2+^ and H_4_TBP Cu^2+^ E2 metabolism catalysation ↑ ROS E2 metabolism-ROS + PDT ↑ ICD Synergy with ICB↑ systemic antitumoral immune response	96.6% tumor growth inhibition 98.3% primary tumor regression and 94.9% in distal tumors inhibition with α-PD-L1 synergy Metastasis regression and long term antitumoral memory effects	[131]
MIL-101/Fe^3+^	ICG-CpG@MOF	ICG and CpG adsorption	>150 nm	Laser PDT and PTT: 808 nm, 1.5 W/cm^2^, 5 min	4T1	HA mediated tumor cells targeting GSH dependent delivery of CpG PDT + PTT + CpG synergy	Tumor disappearance 18 days after treatment Metastasis inhibition	[132]
MIL-101/Fe^3+^	MMH-NPs	MTO encapsulation + HA coating	173.9 ± 3.7 nm	Laser PTT: 671 nm, 1.0 W/cm^2^, 5 min	CT26	HA mediated tumor cells targeting PTT + chemotherapy ↑ICD and tumor antigen presentation αOX40 administration ↓ immunosuppressive cells	Tumor growth inhibition Abscopal effects and metastasis inhibition	[133]
MIL-100/Fe^3+^	OIMH NPs	ICG and OXA encapsulation + HA coating	127 nm	Laser PTT: 808 nm, 0.8 W/cm^2^, 10 min	CT26	PTT + chemotherapy ↑ICD and antitumor response -Synergy with ICB	Inhibition of primary and distant tumors	[134]
UCS/ Zr^4+^	TPZ/UCS	UCNPs core + CA coating + porphyrin MOF shell + TPZ encapsulation	38–65 nm	Laser PTT: 980 nm, 1.2 W/cm^2^, 20 min (5 min interval every 1 min of irradiation)	CT26	UCNP energy transference to the MOF porphyrin shell↑ ROS production pH dependent release and hypoxia activation of TPZ ↑ ROS production PDT+ TPZ ↑ICD and antitumor immunity Synergy with ICB	Complete tumor suppression Abscopal effects in synergy with α-PD-L1	[135]
TBC-Hf/Hf^4+^	IDOi@TBC-Hf	IDOi encapsulation	83.2 nm	LED PDT: 650 nm, 0.1 W/cm^2^, 15 min	CT26 and MC38	PDT ↑ICD in primary tumors IDO inhibition by IDOi ↓ immunosuppressive TME	Near elimination of the primary tumors Abscopal effect	[136]
pMOF/Zr^4+^	Apt/PDGs-s@pMOF	PDG adsorption + ROS-sensitive crosslinking + Periostin- targeting Apt coating.	96.96 nm	Laser PDT: 660 nm, 0.3 W/cm^2^, 5 min	4T1	Periotin-targeting Apt targeting of tumor cells Deeper penetration of the PDG by crosslinking destruction ↓ intratumoral MDSCs PDT↑ICD a systemic immune response	Primary tumor proliferation inhibition Abscopal effect in distant tumors	[137]
Zr-MOF/Zr^4+^	SNPs@Zr-MOF@RB	PAA coating of SNP core-shell + Zr ions and TCCP adsorption for in situ growth + RB incorporation	≈30 nm	Soft X-ray light for 5 min	4T1	Energy transfer from SNP to the MOF + RB ↑ROS production and ↑deep tissue PDT ↑ICD + deep tissue anti tumor immune response ↓ immunosuppressive TME	Tumor growth inhibition	[138]
Zr-MOF/Zr^4+^	NaLnF_4_@MOF	NaLnF_4_ NPs DHCA modification + growth of the Zr-MOF around NaLnF_4_	36.6 ± 2.2 nm	Light PDT: 980 nm, 0.61 W/cm^2^, 10 min (3 min break for each minute)	CT26	UCL from NaLnF_4_ to MOF ↑ PDT PDT-induced ICD synergises with ICB	Complete eradication of primary tumors 95% tumor inhibition Distant tumor suppression in synergy with α-PD-L1	[139]
PBNP/Fe^3+^ and Fe^2+^	PBNP	________	≈60–90 nm	Laser PTT: 808 nm, 1.875 W/cm^2^, 10 min	Neuro2a	PTT↑ICD and antigen presentation Synergize with ICB	Primary tumor shrinkage Suppression and elimination of primary tumors in synergy with aCTLA-4 Rechallenging tumors eradication	[140]
PBNP/Fe^3+^ and Fe^2+^	CpG- PBNP	CpG adsorption	100–1000 nm	Laser PTT: 808 nm, 1.5 W/cm^2^, 10 min	9464D	PTT↑ICD and antigen presentation PDT+ CpG↑DCs maturation	Complete tumor regression 100% survival rate after 80 days Slower distant tumor growth Rechallenged tumor regression 80% mice survival rate after 125 days	[141]
PBNP/Fe^3+^ and Fe^2+^	CpG- PBNP	CpG adsorption	100–1000 nm	Laser PTT: 808 nm, 0.75 W/cm^2^, 10 min	Neuro2a	PTT↑ICD and antigen presentation PDT+ CpG↑DCs maturation and ↑ synergy with ICB	Primary and distant tumor regression Fast elimination of rechallenged tumors	[142]
PBNP/Fe^3+^ and Fe^2+^	SP94-PB-SF- Cy5.5	SP94 adsorption +SF encapsulation + Cy5.5 adsorption	90–110 nm	Laser PTT: 808 nm, 1.5 W/cm^2^, 10 min	HepG2 and Hepa1-6	SP94 selectively targets HCC cells PTT + SF ↑ICD Synergy with ICB ↑ immune response	100% tumor inhibition 80% mice survival rate Primary, distant and rechallenged tumors suppression in synergy with ICB	[143]
PBNP/Fe^3+^ and Fe^2+^	PBNP	________	51 nm	Laser PTT: 808 nm, 2 W/cm^2^, 10 min	SM1	PTT- induced ICD + aCD137 ICB ↑ antitumoral response	Primary tumor elimination 60% distant tumor growth inhibition 66% rechallenged tumor rejection	[144]

1-MT, 1-methyltryptophan; ACF, acriflavine; aCTLA-4, cytotoxic T-lymphocyte-associated protein 4 antibodies; Apt, aptamer; CA, citric acid; CCCP, Carbonyl cyanide 3-chlorophenyl-hydrazone; Cdk4, Cyclin-dependent kinase 4; CpG, Cytosine-phosphate-Guanine; Cy5.5, cyanine5.5; DAMPs, Damage-associated molecular patterns; DCs, dendritic cells; DHCA, dihydroxyphenylpropionc acid; DOX, doxorubicin; FM, Fused cell cytomembrane; GEM, gemcitabine; GSH, glutathione; HA, Hyaluronic acid; HCC, Hepatocellular carcinoma; HIF-1α, Hypoxia-Inducible Factor; ICB, immune checkpoint blockade; ICD, immunogenic cell death; ICG, indocyanine green; IDOi, Indoleamine 2,3-dioxygenase inhibitor; IMQ, imiquimod; IR820, Indocyanine green; LED, light emitting diode; MAN, mannan; MIL, Material Institute Lavoisier; MOF, metal-organic frameworks; MTO, mitoxantrone; OXA, oxaliplatin; PAA, polyacrylic acid; PAH, polyallylamine hydrochloride; PBNPs, Prussian blue nanoparticles; PCN, Porous Coordination Network; PDA, polydopamine; PDG, GEM-loaded DGLs shells; PD-L1, Programmed death-ligand 1; PDT, photodynamic therapy; PpIX, protoporphyrin IX; PTT, photothermal therapy; R837, Vaccigrade Imiquimod; RB, rose bengal; ROS, reactive oxygen species; SF, sorafenib; siCdk4, small interfering Cyclin-dependent kinase 4; SNPs, scintillator nanoparticles; TAAs, tumor-associated antigens; TBP, tetrabenzoporphyrin; TPZ, tirapazamine; UCNP, lanthanide-doped upconversion nanoparticles; ZIF, Zeolitic imidazolate frameworks.

**Table 2:** Overall summary of the strategies employed in the reviewed studies.

Therapeutic Modality	Most Common MOFs	Most Common Modifications	Overall Immunotherapeutic Approaches	Best Therapeutic Outcomes In Vivo
PDT + immunotherapy	Porphyrin-based MOFs	Tumor cell membrane coating CpG adsorption	ICD through ROS production Immune adjuvants ↑DC maturation ↓TME immunosupression + immune checkpoints inhibition	Erradication of primary tumors 95% distant tumor inhibition
PTT + immunotherapy	ZIF-8, MIL-100 and PBNPs	HA coating CpG adsorption ICG encapsulation	Robust ICD induction ↑DC maturation ↓TME immunosupression + immune checkpoints inhibition	97.7% tumor inhibition Primary tumor erradication in 40% of the mice Nearly no recurrence or metastasis
PTT + PDT + immunotherapy	ZIF-8 and MIL-101	CpG adsorption	Robust ICD induction Immune adjuvants ↑DC maturation	100% primary tumor inhibition and regression No recurrence or metastasis

CpG, Cytosine-phosphate-Guanine; DC, dendritic cell; HA, hyaluronic acid; ICD, immunogenic cell death; ICG, indocyanine green; MIL, Material Institute Lavoisier; MOFs, metal-organic frameowrks; PBNP, Prussian blue nanoparticle; PDT, photodynamic therapy; PTT, photothermal therapy; ROS, reactive oxygen species; TME, tumor microenvironment; ZIF, Zeolitic imidazolate framework.

## Data Availability

Not applicable.
